# 

**Published:** 1894-12

**Authors:** 


### INDEX TO VOLUME XV.

A.

Academy of Stomatology, 729.
A Case of drawing the Lower Jaw Forward, 353.
Advantages and Disadvantages of Specialism, 9.
A Good Appointment, 737.
A Half-Century of Anæsthesia, 341.
Allan, Charles F., Eclecticism the Best Conservative Practice, 294.
Allan, George S. Pyorrhœa Alveolaris—The Other View of it, 219.
Alloys as Nostrums and as Subjects for Investigation, 211.
Amalgam Fillings, Injurious Effects of, 81.
Amalgam *versus* Gold, 315.
A Manual of Dental Anatomy, Human and Comparative, 665.
American Academy of Dental Science, 263, 331, 380, 455, 514, 768.
American Dental Association, 479, 720.
American Dental Society of Europe, 479, 657.
American Dentistry in London, 313.
A Modern Wizard, 667.
Anæsthesia, A Half-Century of, 341.
Anæsthetics in Dental Surgery, Notes on, 74.
An Artificial Nose, 609.
Andrews, R. R. Review of Professor Peirce's Paper, Pyorrhœa Alveolaris, 242.
A New Era in Dental Practice, 562.
A New Form of Rubber Wedge, 368.
An Illustrated Dictionary of Medicine, Biology, and Allied Sciences, 602.
An Interesting Case of Exostosis; Asepsis in Operations, 485.
Annual Meeting of Harvard Odontological Society, 286.
Annual of the Universal Medical Sciences, 76.
An Obtundent for Sensitive Dentine, 202.
An Operation, Report of, 253.
Another Advance is Necessary, 194.
Antidote for Arsenic, 207.
  Correction of Formula, 346.
Are our Dental Organizations Satisfactory? 129.
A Reply to Dr. Kirk's Article on Coagulation, 509.
Are we degenerating? 662.
Asepsis in Operations; an Interesting Case of Exostosis, 485.
A Simple Test for Cocaine in Local Anæsthetics, 437.
A Substitute for Copper Amalgam, 319.
A Treatise on Pyorrhœa Alveolaris; a System for its Prompt, Positive, and Permanent Cure, 666.
A Word concerning Discussion and Criticism, 607.

B.

Bell, Victor C. Popular Essays upon the Care of the Teeth and Mouth, 283.
Best Conservative Practice, Eclecticism the, 294.
Bibliography: A Manual of Dental Anatomy, Human and Comparative, 665.
  A Modern Wizard, 667.
  Anæsthetics in Dental Surgery, Notes on, 74.
  An Illustrated Dictionary of Medicine, Biology, and Allied Sciences, 602.
  Annual of the Universal Medical Sciences, 76.
  A Treatise on Pyorrhœa Alveolaris; a System for its Prompt, Positive, and Permanent Cure, 666.
  Bell, Victor C. Popular Essays upon the Care of the Teeth and Mouth, 283.
  Bödecker, C. F. W. The Anatomy and Pathology of the Teeth, 793.
  Catching's Compendium of Practical Dentistry for 1893, 282.
  Chemistry and Physics, 75.
  Cravens, Junius E. A Treatise on Pyorrhœa Alveolaris; a System for its Prompt, Positive, and Permanent Cure, 666.
  Diseases and Injuries of the Teeth, including Pathology and Treatment, 133.
  Gallaudet, Bern B. Surgery, 75.
  Gould, George M. An Illustrated Dictionary of Medicine, Biology, and Allied Sciences, 602.
  Nevins, Laird W. The Discovery of Modern Anæsthesia. By whom was it made? A Brief Statement of Facts, 478.
  Notes on Anæsthetics in Dental Surgery, 74.
  Ottolengui, R. A Modern Wizard, 667.
  Pearson's Dentists' Appointment Book, 198.
  Physics and Chemistry, 75.
  Popular Essays upon the Care of the Teeth and Mouth, 283.

Bibliography:
Smale, Morton. Diseases and Injuries of the Teeth, including Pathology and Treatment, 133.
Struthers, Joseph. Chemistry and Physics, 75.
Surgery, 75.
Talbot, Eugene S. The Etiology of Osseous Deformities of the Head, Face, Jaws, and Teeth, 410.
The Anatomy and Pathology of the Teeth, 793.
The *Dental Cosmos* Calendar, 198.
The Discovery of Modern Anæsthesia. By whom was it made? A Brief Statement of Facts, 478.
The Etiology of Osseous Deformities of the Head, Face, Jaws, and Teeth, 410.
Tomes, Charles S. A Manual of Dental Anatomy, Human and Comparative, 665.
Underwood, Arthur S. Notes on Anæsthetics in Dental Surgery, 74.
Universal Medical Sciences, Annual of, 76.
Ward, D. W. Chemistry and Physics, 75.
Willmarth, Charles H. Chemistry and Physics, 75.
Bödecker, C. F. W. The Anatomy and Pathology of the Teeth, 793.
Bonwill, W. G. A. A New Era in Dental Practice, 562.
Hypnotism, Animal Magnetism, and Personal Magnetism as applied to Dentistry, 21.
Broomell, I. Norman. Metallic *versus* The Plastic Base for Artificial Dentures, 620.
Brubaker, Albert P. Comments on Professor Peirce's Paper, Pyorrhœa Alveolaris, 249.
The Causation of Dental Erosion, 739.
Burchard, Henry. The Editorial Function, 624.

C.

Care and Preservation of Eyesight, 413.
Case in Practice, 347.
Catching's Compendium of Practical Dentistry for 1893, 282.
Central Dental Association of Northern New Jersey, 64, 287, 467.
Cervical Cavities and their Treatment, 758.
Change in the Date of the New Jersey July Examinations, 350.
Change of Name, 738.
Chemical Researches on the Mineral Matter of Bone and Teeth, 802.
Chemistry and Physics, 75.
Chicago Dental Society, 414.
Coagulants: A Further Reply to Dr. Kirk, 706.
Cohesiveness of Tin Fillings, 443.
College Advertising, 201.
Combining Rubber Filings with Fresh Rubber, 255.
Comments on Professsor Peirce's Paper, Pyorrhœa Alveolaris, 249, 251.
Connecticut State Dental Association, 528.
Connecticut Valley Dental Society, 415.
Contours, Porcelain, 309.
Cravens, Junius E. A Treatise on Pyorrhœa Alveolaris; a System for its Prompt, Positive, and Permanent Cure, 666.
Creosotal, 207.
Crown- and Bridge-Work, 303.
Cryer, M. H. The Technique of Nerve Resections for the Relief of Pain about the Face and Jaws, 699.
Current News: American Dental Association, 479, 720.
American Dental Society of Europe, 479, 657.
Annual Meeting of Harvard Odontological Society, 286.
Change in the Date of the New Jersey July Examinations, 350.
Change of Name, 738.
Chicago Dental Society, 414.
Connecticut Valley Dental Society, 415.
Dental Commissioners of Connecticut, 141.
Dental Society of the State of New York, 285.
Dr. Jesse C. Green honored, 78.
First District Dental Society of Illinois, 608.
First District Dental Society, State of New York, 415.
Honored, Dr. Jesse C. Green, 78.
Illinois State Dental Society, 206, 480.
International Medical Congress, 206.
Joint Meeting of the Iowa and Nebraska State Dental Societies, 349.
Massachusetts Dental Society, 414.
Midwinter Fair Dental Congress, 348.
Missouri State Dental Association, 608.
New England Dental Society, 670.
New Jersey State Dental Society, 204.
Ohio State Dental Society, 205, 801.
Pennsylvania State Dental Examining Board, 414.
Recent Patents, 140, 206, 350, 540, 670.
Special Notice—Meeting of the American Dental Association at Old Point Comfort, August 7, 480.
St. Louis Dental Society, 141, 205.
The General Medical Congress of Great Britain, 203.
The Horace Wells Anniversary Celebration, 737.
Transactions of the World's Columbian Dental Congress, 287.
Union Dental Convention, 671.
Woman's Dental Association of the United States, 140, 205, 352, 539, 801.
Cutter, H. E. A Case of drawing the Lower Jaw Forward, 353.

D.

Darby, Edwin T. Comments on Professor Peirce's Paper, Pyorrhœa Alveolaris, 251.
Davenport, S. E. Report of an Operation, 253.
Davenport, Wm. S. Latch-Lock, Lever-Clasp, Plates, and Bridges, 441.
Dental Commissioners of Connecticut, 141.
Dental Erosion, The Causation of, 739.
Dental Instruments, Some Details as to the Care of, 754.
Dental Services to the Insane, 138.
Dental Society of the State of New York, 285.
Deterioration of Vital Energies of Dentists, Some of the Causes of, 289.
Development of Character, 607.
Diet: Its Relation to Tooth-Tissue, 366.
Diplomas in Absentia still in Demand, 72.
Diseases and Injuries of the Teeth, including Pathology and Treatment, 133.
Diseases of the Gums: Their Causes and Treatment, 34.
Domestic Correspondence: Antidote for Arsenic, Correction of Formula, 346.
Case in Practice, 347.
Oristry: Another Name, 606.
Pulp-Nodules, 199.
Reply to Editorial in the *Dental Cosmos*, 533.
Sponge-Grafting, 411.
Dr. Daniel Neall, Obituary, 135.
Dr. Harlan Retires, 133.
Dr. Jesse C. Green honored, 78.
Dr. J. W. Rhone, Obituary, 532.
Dr. Ransom Walker, Obituary, 668.
Dr. Samuel J. Dickey, Obituary, 198.
Duplex Obturator, 545.

E.

Electicism, the Best Conservative Practice, 294.
Editorial: A Good Appointment, 737.
A Half-Century of Anæsthesia, 341.
Another Advance is Necessary, 194.
Are our Dental Organizations Satisfactory? 129.
Are we degenerating? 662.
Diplomas in Absentia still in Demand, 72.
Dr. Harlan Retires, 133.
Medical Criticism, 276.
No more need apply, 197.
No Sex in Science, 602.
Principle in Practice, 530.
Professor W. H. Eames, 343.
Professor W. D. Miller, 281.
Pyorrhœa Alveolaris, 281.
Registering Diplomas in Pennsylvania, 73.
Shall the Entrance Standard be raised? 732.
The Annual Conventions, 477.
The Horace Wells Anniversary Celebration, 736.
The One Discredited Material, 406.
Editorial:
The Opening Year, 70.
The Present Status of Dental Knowledge in the Medical Profession, 598.
The Recent National Conventions, 595.
The Semi-Centennial of the Discovery of Anæsthesia, 478.
Thoughts, Past and Present, 789.
Two Good Papers, 73.
Will We be known by Another Name? 475.
Elevation of Teeth in the Sockets, 417.
Etiology of Pyorrhœa Alveolaris, 1.
Excellence, 77.
Extraction of Teeth to prevent Decay, Some Observations on, 481.

F.

Farlow, John W. On Some Relations of Diseases of Nose and Throat to Dentistry, 491.
Farrar, J. N. Elevation of Teeth in the Sockets, 417.
Physiological and Pathological Changes in the Alveolar Tissues, from Operations for Correction of Irregularities of Teeth, 172.
Some of the Causes of Deterioration of Vital Energies of Dentists, 289.
The Scallop Wire Plate for retaining Teeth in Place after widening the Dental Arch, and for correcting Slight Irregularities when Necessary, 145, 209.
Faught, L. Ashley. Diet: Its Relation to Tooth-Tissue, 366.
First District Dental Society of Illinois, 608.
First District Dental Society, State of New York, 415.
Fish, Wm. L. Cervical Cavities and their Treatment, 758.
Foods, 163.
Foreign Correspondence: London Letter, 797.
Formaline: An Ideal Harmless Germicide, 142.
Francis, Charles E. Cohesiveness of Tin Fillings, 443.
Soft Foils and Plastic Stoppings, 356.
Further Remarks on Pyorrhœa Alveolaris, 501.

G.

Gallaudet, Bern B. Surgery, 75.
Gould, George M. An Illustrated Dictionary of Medicine, Biology, and Allied Sciences, 602.
Graham, Douglass. Massage in Rheumatism of the Temporo-Maxillary Articulation and Muscles of the Lower Jaw, 487.
Grant, George F. Porcelain Contours, 309.
Greenbaum, Max. A Reply to Dr. Kirk's Article on Coagulation, 509.
Coagulants: A Further Reply to Dr. Kirk, 706.
The One Material, 550.

Greenleaf, R. W. Foods, 163.
Guaiacol, 671.
Guilford, S. H. Review of Professor Peirce's Paper, Pyorrhœa Alveolaris, 244.
Gutta-Percha as a Filling-Material, 709.

H.

Harvard Odontological Society, 118, 187, 403, 524, 651, 711, 786.
Headaches, Ocular, 431.
Hemorrhage after Extraction of Teeth in Hemorrhagic Diathesis and Spontaneous Anæmia, 629.
Homœopathic Treatment of Toothache from Pulp-Capping, 495.
Honored, Dr. Jesse C. Green, 78.
Howe, J. Morgan. Alloys as Nostrums and as Subjects for Investigation, 211.
How to Rest, 77.
Human Mouth, The Micro-organisms of the, 285.
Hypnotism, Animal Magnetism, and Personal Magnetism as Applied to Dentistry, 21.

I.

Illinois State Dental Society, 206, 480.
Incidents connected with the Trial of Professor Webster, 41.
Infallibility, 202.
Injurious Effects of Amalgam Fillings, 81.
Interesting and Rare Case, 230.
International Medical Congress, 206.
Is Lysol Poisonous? 543.
Is Prosthetic Dentistry lagging? No, 761.

J.

Jack, Louis. Technique of Conservative Pulp-Treatment, 673.
Joint Meeting of the Iowa and Nebraska State Dental Societies, 349.

K.

Keyes, Charles. The Dental Pulp, Abscesses, and Root-Filling, 499.
Kirk, Edw. C., D.D.S. The Laboratory Method in Dental Education, 746.

L.

Laboratory Methods in Dental Education, 746.
Latch-Lock, Lever-Clasp, Plates, and Bridges, 441.
London Letter, 797.
Lord, Benjamin. Tin-Foil as a Filling-Material, 363.

M.

Massachusetts Dental Society, 414.
Massage in Rheumatism of the Temporo-Maxillary Articulation and Muscles of the Lower Jaw, 487.
Medical Criticism, 276.
Mercuric Chloride, 416.
Metallic *versus* the Plastic Base for Artificial Dentures, 620.
Method of manipulating Gold as practised Fifty Years Ago, 627.
Midwinter Fair Dental Congress, 348.
Mills, George A. Amalgam *versus* Gold, 315.
Missouri State Dental Association, 608.
Mixing Amalgam, 137.
Moriarty, P. W. An Artificial Nose, 609.

N.

National Association of Dental Examiners, 658.
National Association of Dental Faculties, 652.
Need of Independent Medical Journals, 543.
Nevins, Laird W. The Discovery of Modern Anæsthesia. By whom was it made? A Brief Statement of Facts, 478.
Newbold, Wm. Romaine. The Psychological Aspect of Hypnosis, 92.
New England Dental Society, 670.
New Jersey State Dental Society, 204.
New York Odontological Society, 44, 105, 256, 320, 368, 444, 575, 633.
Noble, Lester. Incidents connected with the Trial of Professor Webster, 41.
No More need apply, 197.
No Sex in Science, 602.
Notes and Comments: An Obtundent for Sensitive Dentine, 202.
  A Word concerning Discussion and Criticism, 607.
  Care and Preservation of Eyesight, 413.
  College Advertising, 201.
  Dental Services to the Insane, 138.
  Development of Character, 607.
  Excellence, 77.
  How to rest, 77.
  Human Mouth, The Micro-organisms of the, 285.
  Infallibility, 202.
  Mixing Amalgam, 137.
  Overwork, 200.
  Pathology of the Alveolar Dental Membrane, 536.
  Plaster of Paris, 537.
  Presence of Mind in applying Antidotes, 200.
  Professional Etiquette, 201.
  Professional Fees, 77, 139.
  Pulp-Capping, 137.
  Self-Teaching, 201.
  Septic Infection from a Bur, 538.
  Some of the Necessities for a Creditable Literature, 139.
  Stimulants and Hemorrhage, 77.
  The Art of Thinking, 412.
  The Assimilation of Minerals, 608.
  The Cause of Hypnosis, 347.
  The Independence of Character, 412.
  The Micro-organisms of the Human Mouth, 285.

Notes and Comments:
The Relative Merits of Filling-Material, 413.
The Successful Dentist, 139.
The Treatment of "Quacks," 138.
Nostrums, Alloys as, and as Subjects for Investigation, 211.
Notes on Anæsthetics in Dental Surgery, 74.

O.

Obituary: Dr. Daniel Neall, 135.
Dr. J. W. Rhone, 532.
Dr. Ransom Walker, 668.
Dr. Samuel J. Dickey, 198.
Resolutions of Respect to Dr. Eames, 411.
Resolutions of Respect to Dr. Winder, 605.
Richard Bayly Winder, M.D., D.D.S., 604.
William Henry Eames, D.D.S., 344.
Ocular Headaches, 431.
Odontological Society of Pennsylvania, 54, 173, 270, 388, 580, 644, 781.
Ohio State Dental Society, 205, 801.
On Some Relations of Diseases of the Nose and Throat to Dentistry, 491.
Original Communications: A Case of drawing the Lower Jaw Forward, 353.
Advantages and Disadvantages of Specialism, 9.
Allan, Charles F. Eclecticism, the Best Conservative Practice, 294.
Allan, George S. Pyorrhœa Alveolaris—The Other View of it, 219.
Alloys as Nostrums and as Subjects for Investigation, 211.
Amalgam Fillings, Injurious Effects of, 81.
Amalgam *versus* Gold, 315.
American Dentistry in London, 313.
An Artificial Nose, 609.
Andrews, R. R. Review of Professor Peirce's Paper, Pyorrhœa Alveolaris, 242.
A New Form of Rubber Wedge, 368.
A New Era in Dental Practice, 562.
An Interesting Case of Exostosis; Asepsis in Operations, 485.
An Operation, Report of, 253.
A Reply to Dr. Kirk's Article on Coagulation, 509.
Asepsis in Operations; an Interesting Case of Exostosis, 485.
A Simple Test for Cocaine in Local Anæsthetics, 437.
A Substitute for Copper Amalgam, 319.
Best Conservative Practice, Eclecticism the, 294.
Bonwill, W. G. A. A New Era in Dental Practice, 562.
Hypnotism, Animal Magnetism, and Personal Magnetism as applied to Dentistry, 21.

Original Communications:
Broomell, I. Norman. Metallic *versus* The Plastic Base for Artificial Dentures, 620.
Brubaker, Albert P. Comments on Professor Peirce's Paper, Pyorrhœa Alveolaris, 249.
Burchard, Henry. The Editorial Function, 624.
Cervical Cavities and their Treatment, 758.
Coagulants: A Further Reply to Dr. Kirk, 706.
Cohesiveness of Tin Fillings, 443.
Combining Rubber Filings with Fresh Rubber, 255.
Comments on Professor Peirce's Paper, Pyorrhœa Alveolaris, 249, 251.
Contours, Porcelain, 309.
Crown- and Bridge-Work, 303.
Cryer, M. H. The Technique of Nerve Resections for the Relief of Pain about the Face and Jaws, 699.
Cutter, H. E. A Case of drawing the Lower Jaw Forward, 353.
Darby, Edwin T. Comments on Professor Peirce's Paper, Pyorrhœa Alveolaris, 251.
Davenport, S. E. Report of an Operation, 253.
Davenport, William S. Latch-Lock, Lever-Clasp, Plates, and Bridges, 441.
Deterioration of Vital Energies of Dentists, Some of the Causes of, 289.
Diet: Its Relation to Tooth-Tissue, 366.
Diseases of the Gums: Their Causes and Treatment, 34.
Duplex Obturator, 545.
Eclecticism, the Best Conservative Practice, 294.
Elevation of Teeth in the Sockets, 417.
Etiology of Pyorrhæa Alveolaris, 1.
Extraction of Teeth to prevent Decay, Some Observations on, 481.
Farlow, John W. On Some Relations of Diseases of the Nose and Throat to Dentistry, 491.
Farrar, J. N. Elevation of Teeth in the Sockets, 417.
Physiological and Pathological Changes in the Alveolar Tissues, from Operations of Correction of Irregularities of Teeth, 172.
Some of the Causes of Deterioration of Vital Energies of Dentists, 289.
The Scallop Wire Plate for retaining Teeth in Place after widening the Dental Arch, and for correcting Slight Irregularities when Necessary, 145, 209.
Faught, L. Ashley. Diet: Its Relation to Tooth-Tissue, 366.
Foods, 163.

Original Communications:
Francis, Charles E. Cohesiveness of Tin Fillings, 443.
Soft Foils and Plastic Stoppings, 356.
Further Remarks on Pyorrhœa Alveolaris, 501.
Graham, Douglass. Massage in Rheumatism of the Temporo-Maxillary Articulation and Muscles of the Lower Jaw, 487.
Grant, George F. Porcelain Contours, 309.
Greenbaum, Max. A Reply to Dr. Kirk's Article on Coagulation, 509.
Coagulants: A Further Reply to Dr. Kirk, 706.
The One Material, 550.
Greenleaf, R. W. Foods, 163.
Guilford, S. H. Review of Professor Peirce's Paper, Pyorrhœa Alveolaris, 244.
Gutta-Percha as a Filling-Material, 709.
Headaches, Ocular, 431.
Hemorrhages after Extraction of Teeth in Hemorrhagic Diathesis and Spontaneous Anæmia, 629.
Homœopathic Treatment of Toothache from Pulp-Capping, 495.
Howe, J. Morgan. Alloys as Nostrums and as Subjects for Investigation, 211.
Hypnotism, Animal Magnetism, and Personal Magnetism as Applied to Dentistry, 21.
Incidents connected with the Trial of Professor Webster, 41.
Injurious Effects of Amalgam Fillings, 81.
Interesting and Rare Case, 230.
Is Prosthetic Dentistry lagging? No, 761.
Jack, Louis. Technique of Conservative Pulp-Treatment, 673.
Keyes, Charles. The Dental Pulp, Abscesses, and Root-Filling, 499.
Latch-Lock, Lever-Clasp, Plates, and Bridges, 441.
Lord, Benjamin. Tin-Foil as a Filling-Material, 363.
Massage in Rheumatism of the Temporo-Maxillary Articulation and Muscles of the Lower Jaw, 487.
Metallic *versus* the Plastic Base for Artificial Dentures, 620.
Method of manipulating Gold as practised Fifty Years Ago, 627.
Mills, George A. Amalgam *versus* Gold, 315.
Moriarty, P. W. An Artificial Nose, 609.
Newbold, Wm. Romaine. The Psychological Aspect of Hypnosis, 92.
Noble, Lester. Incidents connected with the Trial of Professor Webster, 41.

Original Communications:
Nostrums, Alloys as, and as Subjects for Investigation, 211.
Ocular Headaches, 431.
On Some Relations of Diseases of the Nose and Throat to Dentistry, 491.
Ottolengui, R. Some Observations upon Pyorrhœa Alveolaris; Review of Professor Peirce's Paper, 230.
Palmer, S. B. Review and Revision of Practice, 610.
Peirce, C. N. Etiology of Pyorrhœa Alveolaris, 1.
Further Remarks on Pyorrhœa Alveolaris, 217, 501.
Perry, Safford G. The Care of the Teeth to the Fifteenth Year, 148.
Physiological and Pathological Changes in the Alveolar Tissues from Operations for Correction of Irregularities of Teeth, 172.
Porcelain Contours, 309.
Porter, Alfred H. The Physiology of Hypnotism, 686.
Pyorrhœa Alveolaris, 30, 217.
Etiology of, 1.
The Other View of it, 219.
Replantation in Chronic Alveolar Abscess, 628.
Report of an Operation, 253.
Review and Revision of Practice, 610.
Review of Professor Peirce's Paper, Pyorrhœa Alveolaris, 230, 236, 242, 244, 247.
Rhein, M. L. Review of Professor Peirce's Paper, Pyorrhœa Alveolaris, 247.
Rhone, J. W. Duplex Obturator, 545.
Rosenthal, D. A. Hemorrhages after Extraction of Teeth in Hemorrhagic Diathesis and Spontaneous Anæmia, 629.
Sabater, D. M. Diseases of the Gums: Their Causes and Treatment, 34.
Simonton, W. S. Combining Rubber Filings with Fresh Rubber, 255.
Smith, Eugene H. Some Observations on the Extraction of Teeth to Prevent Decay, 481.
Smith, F. B. Method of manipulating Gold as practised Fifty Years Ago, 627.
Smith, H. Carleton. A Simple Test for Cocaine in Local Anæsthetics, 437.
Soft Foils and Plastic Stoppings, 356.
Some Details as to the Care of Dental Instruments, 754.
Some Observations on the Extraction of Teeth to prevent Decay, 481.
Some Observations upon Pyorrhœa Alveolaris; Review of Professor Peirce's Paper, 230.
Some of the Causes of Deterioration of Vital Energies of Dentists, 289.
Specialism, Advantages and Disadvantages of, 9.

Original Communications:
Stacey, Charles F. Ocular Headaches, 431.
Stoddard, Arthur H. Gutta-Percha as a Filling-Material, 709.
Sullivan, Geo. A. Replantation in Chronic Alveolar Abscess, 628.
Taft, Charles H. Homœopathic Treatment of Toothache from Pulp-Capping, 495.
Injurious Effects of Amalgam Fillings, 81.
Talbot, E. S. Interesting and Rare Case, 230.
Technique of Conservative Pulp Treatment, 673.
The Care of the Teeth to the Fifteenth Year, 148.
The Causation of Dental Erosion, 739.
The Dental Pulp, Abscesses, and Root-Filling, 499.
The Editorial Function, 624.
The History and Clinical Appearances of Syphilis, with Especial Reference to the Dangers arising from its Oral Manifestations, 421.
The Laboratory Method in Dental Education, 747.
The One Material, 550.
The Other View of it—Pyorrhœa Alveolaris, 219.
The Pathology and Treatment of Pulpless Teeth, 690.
The Physiology of Hypnotism, 686.
The Psychological Aspect of Hypnosis, 92.
The Scallop Wire Plate for retaining Teeth in Place after widening the Dental Arch, and for correcting Slight Irregularities when Necessary, 145, 209.
The Technique of Nerve Resections for the Relief of Pain about the Face and Jaws, 699.
Thomas, Reuen. The Advantages and Disadvantages of Specialism, 9.
Tin-Foil as a Filling-Material, 363.
Trial of Professor Webster, Incidents connected with, 41.
Trueman, William H. A Substitute for Copper Amalgam, 319.
Is Prosthetic Dentistry lagging? No, 171.
Truman, James. Review of Professor C. N. Peirce's Recent Conclusions in Regard to Pyorrhœa Alveolaris, 236.
Van Woert, F. T. Pyorrhœa Alveolaris, 30.
Westlake, Albert. An Interesting Case of Exostosis; Asepsis in Operations, 485.
White, W. H. The Pathology and Treatment of Pulpless Teeth, 690.
Whitney, Charles M. The History and Clinical Appearances of Syphilis, with Especial Reference to the Dangers arising from its Oral Manifestations, 421.
Wilkinson, Frank M. American Dentistry in London, 313.
Wilson, Cecil P. Crown- and Bridge-Work, 303.
Oristry: Another Name, 606.
Ottolengui, R. A Modern Wizard, 667.
Some Observations upon Pyorrhœa Alveolaris; Review of Professor Peirce's Paper, 230.
Overwork, 200.

P.

Palmer, S. B. Review and Revision of Practice, 610.
Pathology of the Alveolar Dental Membrane, 536.
Pearson's Dentists' Appointment-Book, 198.
Peirce, C. N. Etiology of Pyorrhœa Alveolaris, 1, 217.
Further Remarks on Pyorrhœa Alveolaris, 501.
Pennsylvania State Dental Examining Board, 414.
Perry, Safford G. The Care of the Teeth to the Fifteenth Year, 148.
Physics and Chemistry, 75.
Physiological and Pathological Changes in the Alveolar Tissues, from Operations for Correction of Irregularities of Teeth, 172.
Plaster of Paris, 537.
Popular Essays upon the Care of the Teeth and Mouth, 283.
Porcelain Contours, 309.
Porter, Alfred H. The Physiology of Hypnotism, 686.
Potter, Wm. H., D.M.D. Some Details as to the Care of Dental Instruments, 754.
Presence of Mind in applying Antidotes, 200.
Principle in Practice, 530.
Professional Etiquette, 201.
Professional Fees, 77, 139.
Professor W. D. Miller, 281.
Professor W. H. Eames, 343.
Prosthetic Dentistry: Is it lagging? No, 761.
Pulp-Capping, 137.
Pulp Nodules, 199.
Pyorrhœa Alveolaris, 30, 217, 281.
Etiology of, 1.
The Other View of it, 219.

R.

Recent Patents, 140, 206, 350, 540, 670.
Registering Diplomas in Pennsylvania, 73.
Replantation in Chronic Alveolar Abscess, 628.
Reply to Editorial in the *Dental Cosmos*, 533.
Report of an Operation, 253.
Reports of Society Meetings: Academy of Stomatology, 729.
American Academy of Dental Science, 263, 331, 380, 455, 514, 768.

Reports of Society Meetings:
Central Dental Association of Northern New Jersey, 64, 287, 467.
Connecticut State Dental Association, 528.
Harvard Odontological Society, 118, 187, 403, 524, 651, 711, 786.
National Association of Dental Examiners, 658.
National Association of Dental Faculties, 652.
New York Odontological Society, 44, 105, 256, 320, 368, 444, 575, 633.
Odontological Society of Pennsylvania, 54, 173, 270, 388, 580, 644, 781.
Society of Alumni, University of Pennsylvania, 527.
Resection of the Inferior Maxilla with Immediate Prosthesis of an Artificial Jaw, 143.
Resolutions of Respect to Dr. Eames, 411.
Resolutions of Respect to Dr. Winder, 605.
Review and Revision of Practice, 610.
Review of Professor C. N. Peirce's Recent Conclusions in Regard to Pyorrhœa Alveolaris, 230, 236, 242, 244, 247.
Rhein, M. L. Review of Professor Peirce's Paper, Pyorrhœa Alveolaris, 247.
Rhone, J. W. Duplex Obturator, 545.
Richard Bayly Winder, M.D., D.D.S., 604.
Rosenthal, D. A. Hemorrhages after Extraction of Teeth in Hemorrhagic Diathesis and Spontaneous Anæmia, 629.

S.

Sabater, D. M. Diseases of the Gums: Their Causes and Treatment, 34.
Selections: Antidote for Arsenic, 207.
Chemical Researches on the Mineral Matter of Bone and Teeth, 802.
Creosotal, 207.
Formaline: An Ideal Harmless Germicide, 142.
Guaiacol, 671.
Is Lysol Poisonous? 543.
Mercuric Chloride, 416.
Need of Independent Medical Journals, 543.
Resection of the Inferior Maxilla with Immediate Prosthesis of an Artificial Jaw, 143.
The Dangers of Aconite, 415.
The Defect in the Dentist, 288.
Thiocamf, 208.
Self-Teaching, 201.
Septic Infection from a Bur, 538.
Shall the Entrance Standard be raised? 732.
Simonton, W. S. Combining Rubber Filings with Fresh Rubber, 255.
Smale, Morton. Diseases and Injuries of the Teeth, including Pathology and Treatment, 133.
Smith, Eugene H. Some Observations on the Extraction of Teeth to prevent Decay, 481.
Smith, F. B. Method of manipulating Gold as practised Fifty Years Ago, 627.
Smith, H. Carlton. A Simple Test for Cocaine in Local Anæsthetics, 437.
Society of Alumni, University of Pennsylnia, 527.
Soft Foils and Plastic Stoppings, 356.
Some Details as to the Care of Dental Instruments, 754.
Some Observations on the Extraction of Teeth to prevent Decay, 481.
Some Observations upon Pyorrhœa Alveolaris; Review of Professor Peirce's Paper, 230.
Some of the Causes of Deterioration of Vital Energies of Dentists, 289.
Some of the Necessities for a Creditable Literature, 139.
Specialism, Advantages and Disadvantages of, 9.
Special Notice.—Meeting of the American Dental Association at Old Point Comfort, August 7, 480.
Sponge-Grafting, 411.
Stacey, Charles F. Ocular Headaches, 431.
Stimulants and Hemorrhage, 77.
St. Louis Dental Society, 141, 205.
Stoddard, Arthur H. Gutta-Percha as a Filling-Material, 709.
Struthers, Joseph. Chemistry and Physics, 75.
Sullivan, George A. Replantation in Chronic Alveolar Abscess, 628.
Surgery, 75.

T.

Talbot, Eugene S. Interesting and Rare Case, 230.
The Etiology of Osseous Deformities of the Head, Face, Jaws, and Teeth, 410.
Taft, Charles H. Homœopathic Treatment of Toothache from Pulp-Capping, 495.
Injurious Effects of Amalgam Fillings, 81.
Technique of Conservative Pulp-Treatment, 673.
The Annual Conventions, 477.
The Art of Thinking, 412.
The Assimilation of Minerals, 608.
The Care of the Teeth to the Fifteenth Year, 148.
The Cause of Hynopsis, 347.
The Dangers of Aconite, 415.
The Defect in the Dentist, 288.
The *Dental Cosmos* Calendar, 198.
The Dental Pulp, Abscesses, and Root-Filling, 499.
The Discovery of Modern Anæsthesia. By whom was it made? A Brief Statement of Facts, 478.
The Editorial Function, 624.
The Etiology of Osseous Deformities of the Head, Face, Jaws, and Teeth, 410.
The General Medical Council of Great Britain, 203.

The History and Clinical Appearances of Syphilis, with Especial Reference to the Dangers arising from its Oral Manifestation, 421.
The Horace Wells Anniversary Celebration, 736.
The Independence of Character, 412.
The Laboratory Method in Dental Education, 746.
The Micro-organisms of the Human Mouth, 285.
The One Discredited Material, 406.
The One Material, 550.
The Opening Year, 70.
The Other View of it—Pyorrhœa Alveolaris, 219.
The Pathology and Treatment of Pulpless Teeth, 690.
The Physiology of Hypnotism, 686.
The Present Status of Dental Knowledge in the Medical Profession, 598.
The Psychological Aspect of Hypnosis, 92.
The Recent National Conventions, 595.
The Relative Merit of Filling-Material, 413.
The Scallop Wire Plate for retaining Teeth in Place after widening the Dental Arch, and for correcting Slight Irregularities when Necessary, 145, 209.
The Semi-Centennial of the Discovery of Anæsthesia, 478.
The Successful Dentist, 139.
The Technique of Nerve-Resections for the Relief of Pain about the Face and Jaws, 699.
The Treatment of "Quacks," 138.
Thiocamf, 208.
Thomas, Reuen. Advantages and Disadvantages of Specialism, 9.
Thoughts, Past and Present, 789.
Tin-Foil as a Filling-Material, 363.
Tomes, Charles S. A Manual of Dental Anatomy, Human and Comparative, 665.
Transactions of the World's Columbian Dental Congress, 287.
Trueman, William H. A Substitute for Copper Amalgam, 319.
Is Prosthetic Dentistry lagging? No, 761.
Truman, James. Review of Professor C. N. Peirce's Recent Conclusions in Regard to Pyorrhœa Alveolaris, 236.
Trial of Professor Webster, Incidents connected with, 4.
Two Good Papers, 73.

U.

Underwood, Arthur S. Notes on Anæsthetics in Dental Surgery, 74.
Union Dental Convention, 671.
Universal Medical Sciences, Annual of, 76.

V.

Van Woert, F. T. Pyorrhœa Alveolaris, 30.

W.

Ward, D. M. Chemistry and Physics, 75.
Westlake, Albert. An Interesting Case of Exostosis; Asepsis in Operations, 485.
White, W. H. The Pathology and Treatment of Pulpless Teeth, 690.
Whitney, Charles M. The History and Clinical Appearances of Syphilis, with Especial Reference to the Dangers arising from its Oral Manifestations, 421.
Wilkinson, Frank M. American Dentistry in London, 313.
William Henry Eames, D.D.S., 344.
Willmarth, Charles H. Chemistry and Physics, 75.
Will We be known by Another Name? 475.
Wilson, Cecil P. Crown- and Bridge-Work, 303.
Woman's Dental Association of the United States, 140, 205, 352, 539, 801.

### LIST OF CONTRIBUTORS TO VOLUME XV.

Allan, Dr. Charles F., Newburgh, N. Y.
Allan, Geo. S., D.D.S., New York.
Andrews, Dr. R. R., Boston, Mass.
Bonwill, W. G. A., D.D.S., Philadelphia.
Broomell, I. Norman, D.D.S., Philadelphia.
Brubaker, A. P., M.D., D.D.S., Philadelphia.
Burchard, Henry, M.D., D.D S., Philadelphia.
Cryer, M. H., M.D., D.D.S., Philadelphia.
Cutter, H. E., D.D.S., Cambridge, Mass.
Darby, Edwin T., M.D., D.D.S., Philadelphia.
Davenport, Dr. S. E., New-York.
Davenport, Wm. S., D.D.S., Paris, France.
Farlow, John W., M.D., Boston, Mass.
Farrar, J. N., M.D., D.D.S., New York.
Faught, L. Ashley, D.D.S., Philadelphia.
Fish, Wm. L., D.D.S., Newark, N. J.
Francis, Dr. C. E., New York.
Graham, Douglas, M.D., Boston.
Grant, Geo. F., D.M.D., Boston.
Greenbaum, Max, D.D.S., Philadelphia.
Greenleaf, Professor R. W.
Guilford, S. H., D.D.S., Philadelphia.
Howe, Dr. J. Morgan, New York.
Jack, Louis, D.D.S., Philadelphia.
Keyes, Charles, D.D S., Rio de Janeiro, Brazil.
Kirk, Edward C., D.D.S., Philadelphia.
Lord, Dr. Benjamin, New York.
Mills, Dr. Geo. A., New York.
Moriarty, P. W., D.M.D., Boston.
Newbold, Wm Romaine, Ph.D., Philadelphia.
Noble, Dr. Lester, Springfield, Mass.
Ottolengui, R., M.D.S., New York.

PALMER, S. B., M.D.S., Syracuse, N. Y.

PEIRCE, C. N., D.D.S., Philadelphia.

PERRY, SAFFORD G., D.D.S., New York.

PORTER, ALFRED H., D.D.S., Philadelphia.

POTTER, WM. H., D.M.D., Boston.

RHEIN, M. L., M.D., D.D.S., New York.

RHONE, DR. J. W., Bellefonte, Pa.

ROLLINS, WILLIAM HERBERT, Boston.

ROSENTHAL, D. A., D.D.S., Philadelphia.

SABATER, D. M., A.B., D.D.S., M.D., New York.

SIMONTON, DR. W. S., West Virginia.

SMITH, EUGENE H., D.M.D., Boston.

SMITH, F. B., M.D., New York.

SMITH, H. CARLTON, Boston.

STACEY, CHARLES F., M.D., Boston.

STODDARD, ARTHUR H., D.M.D., Boston.

SULLIVAN, GEO. A., D.D.S., Albany, N. Y.

TAFT, CHARLES H., A.B., D.M.D., Chicago.

TALBOT, E. S., D.D.S., Chicago.

THOMAS, REUEN, D.D., PH.D., Brookline, Mass.

TRUEMAN, WM. H., D.D.S., Philadelphia.

TRUMAN, JAMES, D.D.S., Philadelphia.

VAN WOERT, DR. F. T., New York.

WESTLAKE, ALBERT, D.D.S., New York.

WHITE, DR. W. H., Silver City, New Mexico.

WHITNEY, CHARLES M., M.D., Boston.

WILKINSON, FRANK M., D.M.D., Boston.

WILSON, CECIL P., D.M.D.

SUBSCRIBE FOR THE

### International Dental Journal,

EDITED BY JAMES TRUMAN, D.D.S.

### Published by the International Dental Publication Company.

LOUIS JACK, PRESIDENT, Philadelphia.
BENJAMIN LORD, VICE-PRESIDENT, New York.
C. N. PEIRCE, TREASURER, Philadelphia.
GEO. S. ALLEN, SECRETARY, 51 West Thirty-seventh St., New York.

**Publication Office, 716 Filbert St., Philadelphia, Pa.**

### STOCKHOLDERS.

| | |
|---|---|
| GEO. S. ALLAN | New York, N.Y. |
| R. R. ANDREWS | Cambridge, Mass. |
| H. A. BAKER | Boston, Mass. |
| A. E. BALDWIN | Chicago, Ill. |
| W. C. BARRETT | Buffalo, N.Y. |
| S. T. BEALE, JR. | Philadelphia, Pa. |
| C. S. BECK | Wilkesbarre, Pa. |
| G. V. BLACK | Jacksonville, Ill. |
| C. F. W. BODECKER | New York, N.Y. |
| E. A. BOGUE | New York, N.Y. |
| W. G. A. BONWILL | Philadelphia, Pa. |
| C. A. BRACKETT | Newport, R.I. |
| THOMAS BRADLEY | New York, N.Y. |
| EDWARD C. BRIGGS | Boston, Mass. |
| ALBERT H. BROCKWAY | Brooklyn, N.Y. |
| A. E. BROWN | Chicago, Ill. |
| WM. CARR | New York, N.Y. |
| GEO. H. CHANCE | Portland, Oregon. |
| G. F. CHENEY | St. Johnsbury, Vt. |
| DWIGHT M. CLAPP | Boston, Mass. |
| JOHN T. CODMAN | Boston, Mass. |
| CHAS. D. COOK | Brooklyn, N.Y. |
| J. N. CROUSE | Chicago, Ill. |
| EDW. T. DARBY | Philadelphia, Pa. |
| H. S. DRAPER | Boston, Mass. |
| WM. H. DWINELLE | New York, N.Y. |
| A. R. EATON | Elizabeth, N.J. |
| ALBERT T. EMERY | Boston, Mass. |
| CHAS. J. ESSIG | Philadelphia, Pa. |
| J. N. FARRER | New York, N.Y. |
| T. FILLEBROWN | Portland, Me. |
| W. B. FINNEY | Baltimore, Md. |
| T. A. FLETCHER | New York, N.Y. |
| M. W. FOSTER | Baltimore, Md. |
| CHAS. E. FRANCIS | New York, N.Y. |
| E. C. FUNDENBERG | Pittsburg, Pa. |
| W. F. FUNDENBERG | Pittsburg, Pa. |
| W. H. FUNDENBERG | Pittsburg, Pa. |
| E. S. GAYLORD | New Haven, Conn. |
| J. P. GERAN | Brooklyn, N.Y. |
| A. H. GILSON | Boston, Mass. |
| S. H. GUILFORD | Philadelphia, Pa. |
| JOHN I. HART | New York, N.Y. |
| O. E. HILL | Brooklyn, N.Y. |
| J. F. P. HODSON | New York, N.Y. |
| J. MORGAN HOWE | New York, N.Y. |
| ROBERT HUEY | Philadelphia, Pa. |
| A. O. HUNT | Iowa City, Iowa. |
| LOUIS JACK | Philadelphia, Pa. |
| V. H. JACKSON | New York, N.Y. |
| C. R. JEFFERIS | Wilmington, Del. |
| W. T. LA ROCHE | Harrington Park, N.J. |
| W. K. LEECH | Philadelphia, Pa. |
| WILBUR F. LITCH | Philadelphia, Pa. |
| J. BOND LITTIG | New York, N.Y. |
| BENJAMIN LORD | New York, N.Y. |
| GEO. W. LOVEJOY | Montreal, Ont. |
| JOHN S. MARSHALL | Chicago, Ill. |
| H. J. MCKELLOPS | St. Louis, Mo. |
| CHARLES MCMANUS | Hartford, Conn. |
| JAMES MCMANUS | Hartford, Conn. |
| DAN'L NEAL MCQUILLAN | Philadelphia, Pa. |
| FREDERIC A. MERRILL | Boston, Mass. |
| HORATIO C. MERIAM | Salem, Mass. |
| W. D. MILLER | Berlin, Germany. |
| GEO. T. MOFFATT | Boston, Mass. |
| W. E. PAGE | Boston, Mass. |
| S. B. PALMER | Syracuse, N.Y. |
| C. B. PARKER | Brooklyn, N.Y. |
| D. D. PEABODY | Stoneham, Mass. |
| C. N. PEIRCE | Philadelphia, Pa. |
| S. G. PERRY | New York, N.Y. |
| W. A. PHREANER | Philadelphia, Pa. |
| CHAS. E. PIKE | Philadelphia, Pa. |
| W. E. PINKHAM | New York, N.Y. |
| W. H. POTTER | Boston, Mass. |
| CHAS. P. PRUYN | Chicago, Ill. |
| H. C. REGISTER | Philadelphia, Pa. |
| Z. T. SAILER | New York, N.Y. |
| L. D. SHEPARD | Boston, Mass. |
| GEO. R. SHIDLE | Pittsburg, Pa. |
| EUGENE H. SMITH | Boston, Mass. |
| B. HOLLY SMITH | Baltimore, Md. |
| H. A. SMITH | Cincinnati, Ohio. |
| S. G. STEVENS | Boston, Mass. |
| W. X. SUDDUTH | Minneapolis, Minn. |
| EUGENE S. TALBOTT | Chicago, Ill. |
| AMBLER TEES | Philadelphia, Pa. |
| J. D. THOMAS | Philadelphia, Pa. |
| JAMES TRUMAN | Philadelphia, Pa. |
| WM. H. TRUEMAN | Philadelphia, Pa. |
| W. W. WALKER | New York, N.Y. |
| I. F. WARDWELL | Stanford, Conn. |
| G. W. WARREN | Philadelphia, Pa. |
| T. S. WATERS | Baltimore, Md. |
| GEO. W. WELD | New York, N.Y. |
| JULIUS G. W. WERNER | Boston, Mass. |
| JACOB L. WILLIAMS | Boston, Mass. |
| R. B. WINDER | Baltimore, Md. |
| C. A. WOODWARD | New York, N.Y. |
| J. A. WOODWARD | Philadelphia, Pa. |
| W. A. WOODWARD | New York, N.Y. |
| W. J. YOUNGER | San Francisco, Cal. |

The officers of the INTERNATIONAL DENTAL PUBLICATION COMPANY serve without salary, the investments of the stockholders merely guarantee the success of the enterprise, ALL PROFITS being devoted to the enlargement or improvement of the INTERNATIONAL DENTAL JOURNAL.

**The support of every dentist having the welfare of his profession at heart is earnestly solicited. Subscription, $2.50 per Annum.**

*THE INTERNATIONAL DENTAL PUBLICATION CO.*

SUBSCRIBE FOR THE

### International Dental Journal,

EDITED BY JAMES TRUMAN, D.D.S.

### Published by the International Dental Publication Company.

LOUIS JACK, PRESIDENT, Philadelphia.
BENJAMIN LORD, VICE-PRESIDENT, New York.
C. N. PEIRCE, TREASURER, Philadelphia.
GEO. S. ALLEN, SECRETARY, 51 West Thirty-seventh St., New York.

**Publication Office, 716 Filbert St., Philadelphia, Pa.**

### STOCKHOLDERS.

Geo. S. Allan . . . . . . . . New York, N.Y.
R. R. Andrews . . . . . . . . . Cambridge, Mass.
H. A. Baker . . . . . . . . . . . Boston, Mass.
A. E. Baldwin . . . . . . . . . . . Chicago, Ill.
W. C. Barrett . . . . . . . . . . Buffalo, N.Y.
S. T. Beale, Jr. . . . . . . . . Philadelphia, Pa.
C. S. Beck . . . . . . . . . Wilkesbarre, Pa.
G. V. Black . . . . . . . . . Jacksonville, Ill.
C. F. W. Bodecker . . . . . . New York, N.Y.
E. A. Bogue . . . . . . . . . . New York, N.Y.
W. G. A. Bonwill . . . . . . . Philadelphia, Pa.
C. A. Brackett . . . . . . . . . . Newport, R.I.
Thomas Bradley . . . . . . . New York, N.Y.
Edward C. Briggs . . . . . . . . Boston, Mass.
Albert H. Brockway . . . . Brooklyn, N.Y.
A. E. Brown . . . . . . . . . . . Chicago, Ill.
Wm. Carr . . . . . . . . . . New York, N.Y.
Geo. H. Chance . . . . . . Portland, Oregon.
G. F. Cheney . . . . . . . . St. Johnsbury, Vt.
Dwight M. Clapp . . . . . . . . Boston, Mass.
John T. Codman . . . . . . . . . Boston, Mass.
Chas. D. Cook . . . . . . . . . Brooklyn, N.Y.
J. N. Crouse . . . . . . . . . . . Chicago, Ill.
Edw. T. Darby . . . . . . . . Philadelphia, Pa.
H. S. Draper . . . . . . . . . . . Boston, Mass.
Wm. H. Dwinelle . . . . . . New York, N.Y.
A. R. Eaton . . . . . . . . . Elizabeth, N.J.
Albert T. Emery . . . . . . . Boston, Mass.
Chas. J. Essig . . . . . . . . . Philadelphia, Pa.
J. N. Farrer . . . . . . . . . New York, N.Y.
T. Fillebrown . . . . . . . . . . . Portland, Me.
W. B. Finney . . . . . . . . . . Baltimore, Md.
T. A. Fletcher . . . . . . . . New York, N.Y.
M. W. Foster . . . . . . . . . . Baltimore, Md.
Chas. E. Francis . . . . . . . New York, N.Y.
E. C. Fundenberg . . . . . . . . Pittsburg, Pa.
W. F. Fundenberg . . . . . . . . Pittsburg, Pa.
W. H. Fundenberg . . . . . . . . Pittsburg, Pa.
E. S. Gaylord . . . . . . . New Haven, Conn.
J. P. Geran . . . . . . . . . . . . Brooklyn, N.Y.
A. H. Gilson . . . . . . . . . . . Boston, Mass.
S. H. Guilford . . . . . . . Philadelphia, Pa.
John I. Hart . . . . . . . . . New York, N.Y.
O. E. Hill . . . . . . . . . . . Brooklyn, N.Y.
J. F. P. Hodson . . . . . . . . New York, N.Y.
J. Morgan Howe . . . . . . . New York, N.Y.
Robert Huey . . . . . . . . Philadelphia, Pa.
A. O. Hunt . . . . . . . . . . Iowa City, Iowa.
Louis Jack . . . . . . . . . . Philadelphia, Pa.
[illegible]. H. Jackson . . . . . . . . New York, N.Y.
C. R. Jefferis . . . . . . . . . Wilmington, Del.
W. T. La Roche . . . . Harrington Park, N.J.
W. K. Leech . . . . . . . . . Philadelphia, Pa.
Wilbur F. Litch . . . . . . . Philadelphia, Pa.
J. Bond Littig . . . . . . . . . New York, N.Y.
Benjamin Lord . . . . . . . . New York, N.Y.
Geo. W. Lovejoy . . . . . . . . Montreal, Ont.
John S. Marshall . . . . . . . . . Chicago, Ill.
H. J. McKellops . . . . . . . . . St. Louis, Mo.
Charles McManus . . . . . . Hartford, Conn.
James McManus . . . . . . . . Hartford, Conn.
Dan'l Neal McQuillan . . . Philadelphia, Pa.
Frederic A. Merrill . . . . . . Boston, Mass.
Horatio C. Meriam . . . . . . . Salem, Mass.
W. D. Miller . . . . . . . . Berlin, Germany.
Geo. T. Moffatt . . . . . . . . . . Boston, Mass.
W. E. Page . . . . . . . . . . . . Boston, Mass.
S. B. Palmer . . . . . . . . . . . Syracuse, N.Y.
C. B. Parker . . . . . . . . . . Brooklyn, N.Y.
D. D. Peabody . . . . . . . . Stoneham, Mass.
C. N. Peirce . . . . . . . . . Philadelphia, Pa.
S. G. Perry . . . . . . . . . . New York, N.Y.
W. A. Phreaner . . . . . . . Philadelphia, Pa.
Chas. E. Pike . . . . . . . . . Philadelphia, Pa.
W. E. Pinkham . . . . . . . . New York, N.Y.
W. H. Potter . . . . . . . . . . . Boston, Mass.
Chas. P. Pruyn . . . . . . . . . . . Chicago, Ill.
H. C. Register . . . . . . . . Philadelphia, Pa.
Z. T. Sailer . . . . . . . . . New York, N.Y.
L. D. Shepard . . . . . . . . . . . Boston, Mass.
Geo. R. Shidle . . . . . . . . . . . Pittsburg, Pa.
Eugene H. Smith . . . . . . . . . . Boston, Mass.
B. Holly Smith . . . . . . . . . Baltimore, Md.
H. A. Smith . . . . . . . . . . Cincinnati, Ohio.
S. G. Stevens . . . . . . . . . . . Boston, Mass.
W. X. Sudduth . . . . . . . Minneapolis, Minn.
Eugene S. Talbott . . . . . . . . . Chicago, Ill.
Ambler Tees . . . . . . . . . Philadelphia, Pa.
J. D. Thomas . . . . . . . . . Philadelphia, Pa.
James Truman . . . . . . . . Philadelphia, Pa.
Wm. H. Trueman . . . . . . . Philadelphia, Pa.
W. W. Walker . . . . . . . . . New York, N.Y.
I. F. Wardwell . . . . . . . . . Stanford, Conn.
G. W. Warren . . . . . . . . Philadelphia, Pa.
T. S. Waters . . . . . . . . . . Baltimore, Md.
Geo. W. Weld . . . . . . . . . New York, N.Y.
Julius G. W. Werner . . . . . Boston, Mass.
Jacob L. Williams . . . . . . . . Boston, Mass.
R. B. Winder . . . . . . . . . . Baltimore, Md.
C. A. Woodward . . . . . . . New York, N.Y.
J. A. Woodward . . . . . . . Philadelphia, Pa.
W. A. Woodward . . . . . . . New York, N.Y.
W. J. Younger . . . . . . . San Francisco, Cal.

The officers of the International Dental Publication Company serve without salary, the investments of the stockholders merely guarantee the success of the enterprise, ALL PROFITS being devoted to the enlargement or improvement of the International Dental Journal.

**The support of every dentist having the welfare of his profession at heart is earnestly solicited. Subscription, $2.50 per Annum.**

*THE INTERNATIONAL DENTAL PUBLICATION CO.*

SUBSCRIBE FOR THE

### International Dental Journal,

EDITED BY JAMES TRUMAN, D.D.S.

### Published by the International Dental Publication Company.

LOUIS JACK, PRESIDENT, Philadelphia.
BENJAMIN LORD, VICE-PRESIDENT, New York.
C. N. PEIRCE, TREASURER, Philadelphia.
GEO. S. ALLAN, SECRETARY, 51 West Thirty-seventh St., New York.

**Publication Office, 716 Filbert St., Philadelphia, Pa.**

STOCKHOLDERS.

| | |
|---|---|
| GEO. S. ALLAN | New York, N.Y. |
| R. R. ANDREWS | Cambridge, Mass. |
| H. A. BAKER | Boston, Mass. |
| A. E. BALDWIN | Chicago, Ill. |
| W. C. BARRETT | Buffalo, N.Y. |
| S. T. BEALE, JR. | Philadelphia, Pa. |
| C. S. BECK | Wilkesbarre, Pa. |
| G. V. BLACK | Jacksonville, Ill. |
| C. F. W. BODECKER | New York, N.Y. |
| E. A. BOGUE | New York, N.Y. |
| W. G. A. BONWILL | Philadelphia, Pa. |
| C. A. BRACKETT | Newport, R.I. |
| THOMAS BRADLEY | New York, N.Y. |
| EDWARD C. BRIGGS | Boston, Mass. |
| ALBERT H. BROCKWAY | Brooklyn, N.Y. |
| A. E. BROWN | Chicago, Ill. |
| WM. CARR | New York, N.Y. |
| GEO. H. CHANCE | Portland. Oregon. |
| G. F. CHENEY | St. Johnsbury, Vt. |
| DWIGHT M. CLAPP | Boston, Mass. |
| JOHN T. CODMAN | Boston, Mass. |
| CHAS. D. COOK | Brooklyn, N.Y. |
| J. N. CROUSE | Chicago, Ill. |
| EDW. T. DARBY | Philadelphia, Pa. |
| H. S. DRAPER | Boston, Mass. |
| WM. H. DWINELLE | New York, N.Y. |
| A. R. EATON | Elizabeth, N.J. |
| ALBERT T. EMERY | Boston, Mass. |
| CHAS. J. ESSIG | Philadelphia, Pa. |
| J. N. FARRER | New York, N.Y. |
| T. FILLEBROWN | Portland, Me. |
| W. B. FINNEY | Baltimore, Md. |
| T. A. FLETCHER | New York, N.Y. |
| M. W. FOSTER | Baltimore, Md. |
| CHAS. E. FRANCIS | New York, N.Y. |
| E. C. FUNDENBERG | Pittsburg, Pa. |
| W. F. FUNDENBERG | Pittsburg, Pa. |
| W. H. FUNDENBERG | Pittsburg, Pa. |
| E. S. GAYLORD | New Haven, Conn. |
| J. P. GERAN | Brooklyn, N.Y. |
| A. H. GILSON | Boston, Mass. |
| S. H. GUILFORD | Philadelphia, Pa. |
| JOHN I. HART | New York, N.Y. |
| O. E. HILL | Brooklyn, N.Y. |
| J. F. P. HODSON | New York, N.Y. |
| J. MORGAN HOWE | New York, N.Y. |
| ROBERT HUEY | Philadelphia, Pa. |
| A. O. HUNT | Iowa City, Iowa. |
| LOUIS JACK | Philadelphia, Pa |
| V. H. JACKSON | New York, N.Y. |
| C. R. JEFFERIS | Wilmington, Del. |
| W. T. LA ROCHE | Harrington Park, N.J. |
| W. K. LEECH | Philadelphia Pa. |
| WILBUR F. LITCH | Philadelphia, Pa. |
| J. BOND LITTIG | New York, N.Y. |
| BENJAMIN LORD | New York, N.Y. |
| GEO. W. LOVEJOY | Montreal, Ont. |
| JOHN S. MARSHALL | Chicago, Ill. |
| H. J. MCKELLOPS | St. Louis, Mo. |
| CHARLES MCMANUS | Hartford, Conn. |
| JAMES MCMANUS | Hartford, Conn. |
| DAN'L NEAL MCQUILLAN | Philadelphia, Pa. |
| FREDERIC A. MERRILL | Boston, Mass. |
| HORATIO C. MERIAM | Salem, Mass. |
| W. D. MILLER | Berlin, Germany. |
| GEO. T. MOFFATT | Boston, Mass. |
| A. L. NORTHROP | New York, N.Y. |
| W. E. PAGE | Boston, Mass. |
| S. B. PALMER | Syracuse, N.Y. |
| C. B. PARKER | Brooklyn, N.Y. |
| D. D. PEABODY | Stoneham, Mass. |
| C. N. PEIRCE | Philadelphia, Pa. |
| S. G. PERRY | New York, N.Y. |
| W. A. PHREANER | Philadelphia, Pa. |
| CHAS. E. PIKE | Philadelphia, Pa. |
| W. E. PINKHAM | New York, N.Y. |
| W. H. POTTER | Boston, Mass. |
| CHAS. P. PRUYN | Chicago, Ill. |
| H. C. REGISTER | Philadelphia, Pa. |
| F. A. ROY | New Orleans, La. |
| Z. T. SAILER | New York, N.Y. |
| L. D. SHEPARD | Boston, Mass. |
| GEO. R. SHIDLE | Pittsburg, Pa. |
| EUGENE H. SMITH | Boston, Mass. |
| B. HOLLY SMITH | Baltimore, Md. |
| H. A. SMITH | Cincinnati, Ohio. |
| S. G. STEVENS | Boston, Mass. |
| W. X. SUDDUTH | Minneapolis, Minn. |
| EUGENE S. TALBOTT | Chicago, Ill. |
| AMBLER TEES | Philadelphia, Pa. |
| J. D. THOMAS | Philadelphia, Pa. |
| JAMES TRUMAN | Philadelphia, Pa. |
| WM. H. TRUEMAN | Philadelphia, Pa. |
| W. W. WALKER | New York, N.Y. |
| I. F. WARDWELL | Stanford, Conn. |
| G. W. WARREN | Philadelphia. Pa. |
| T. S. WATERS | Baltimore, Md. |
| GEO. W. WELD | New York, N.Y. |
| JULIUS G. W. WERNER | Boston, Mass. |
| JACOB L. WILLIAMS | Boston, Mass. |
| R. B. WINDER | Baltimore, Md. |
| C. A. WOODWARD | New York, N.Y. |
| J. A. WOODWARD | Philadelphia, Pa. |
| W. A. WOODWARD | New York, N.Y. |
| W. J. YOUNGER | San Francisco. Cal. |

The officers of the INTERNATIONAL DENTAL PUBLICATION COMPANY serve without salary, the investments of the stockholders merely guarantee the success of the enterprise, ALL PROFITS being devoted to the enlargement or improvement of the INTERNATIONAL DENTAL JOURNAL.

**The support of every dentist having the welfare of his profession at heart is earnestly solicited. Subscription, $2.50 per Annum.**

*THE INTERNATIONAL DENTAL PUBLICATION CO.*

SUBSCRIBE FOR THE

### International Dental Journal,

EDITED BY JAMES TRUMAN, D.D.S.

### Published by the International Dental Publication Company.

LOUIS JACK, PRESIDENT, Philadelphia.
BENJAMIN LORD, VICE-PRESIDENT, New York.
C. N. PEIRCE, TREASURER, Philadelphia.
GEO. S. ALLAN, SECRETARY, 51 West Thirty-seventh St., New York.

**Publication Office, 716 Filbert St., Philadelphia, Pa.**

STOCKHOLDERS.

| | |
|---|---|
| GEO. S. ALLAN | New York, N.Y. |
| R. R. ANDREWS | Cambridge, Mass. |
| H. A. BAKER | Boston, Mass. |
| A. E. BALDWIN | Chicago, Ill. |
| W. C. BARRETT | Buffalo, N.Y. |
| S. T. BEALE, JR. | Philadelphia, Pa. |
| C. S. BECK | Wilkesbarre, Pa. |
| G. V. BLACK | Jacksonville, Ill. |
| C. F. W. BODECKER | New York, N.Y. |
| E. A. BOGUE | New York, N.Y. |
| W. G. A. BONWILL | Philadelphia, Pa. |
| C. A. BRACKETT | Newport, R.I. |
| THOMAS BRADLEY | New York, N.Y. |
| EDWARD C. BRIGGS | Boston, Mass. |
| ALBERT H. BROCKWAY | Brooklyn, N.Y. |
| A. E. BROWN | Chicago, Ill. |
| WM. CARR | New York, N.Y. |
| GEO. H. CHANCE | Portland, Oregon. |
| G. F. CHENEY | St. Johnsbury, Vt. |
| DWIGHT M. CLAPP | Boston, Mass. |
| JOHN T. CODMAN | Boston, Mass. |
| CHAS. D. COOK | Brooklyn, N.Y. |
| J. N. CROUSE | Chicago, Ill. |
| EDW. T. DARBY | Philadelphia, Pa. |
| H. S. DRAPER | Boston, Mass. |
| WM. H. DWINELLE | New York, N.Y. |
| A. R. EATON | Elizabeth, N.J. |
| ALBERT T. EMERY | Boston, Mass. |
| CHAS. J. ESSIG | Philadelphia, Pa. |
| J. N. FARRER | New York, N.Y. |
| T. FILLEBROWN | Portland, Me. |
| W. B. FINNEY | Baltimore, Md. |
| T. A. FLETCHER | New York, N.Y. |
| M. W. FOSTER | Baltimore, Md. |
| CHAS. E. FRANCIS | New York, N.Y. |
| E. C. FUNDENBERG | Pittsburg, Pa. |
| W. F. FUNDENBERG | Pittsburg, Pa. |
| W. H. FUNDENBERG | Pittsburg, Pa. |
| E. S. GAYLORD | New Haven, Conn. |
| J. P. GERAN | Brooklyn, N.Y. |
| A. H. GILSON | Boston, Mass. |
| S. H. GUILFORD | Philadelphia, Pa. |
| JOHN I. HART | New York, N.Y. |
| O. E. HILL | Brooklyn, N.Y. |
| J. F. P. HODSON | New York, N.Y. |
| J. MORGAN HOWE | New York, N.Y. |
| ROBERT HUEY | Philadelphia, Pa. |
| A. O. HUNT | Iowa City, Iowa. |
| LOUIS JACK | Philadelphia, Pa |
| V. H. JACKSON | New York, N.Y. |
| C. R. JEFFERIS | Wilmington, Del. |
| W. T. LA ROCHE | Harrington Park, N.J. |
| W. K. LEECH | Philadelphia, Pa. |
| WILBUR F. LITCH | Philadelphia, Pa. |
| J. BOND LITTIG | New York, N.Y. |
| BENJAMIN LORD | New York, N.Y. |
| GEO. W. LOVEJOY | Montreal, Ont. |
| JOHN S. MARSHALL | Chicago, Ill. |
| H. J. MCKELLOPS | St. Louis, Mo. |
| CHARLES MCMANUS | Hartford, Conn. |
| JAMES MCMANUS | Hartford, Conn. |
| DAN'L NEAL MCQUILLAN | Philadelphia, Pa. |
| FREDERIC A. MERRILL | Boston, Mass. |
| HORATIO C. MERIAM | Salem, Mass. |
| W. D. MILLER | Berlin, Germany. |
| GEO. T. MOFFATT | Boston, Mass. |
| A. L. NORTHROP | New York, N.Y. |
| W. E. PAGE | Boston, Mass. |
| S. B. PALMER | Syracuse, N.Y. |
| C. B. PARKER | Brooklyn, N.Y. |
| D. D. PEABODY | Stoneham, Mass. |
| C. N. PEIRCE | Philadelphia, Pa. |
| S. G. PERRY | New York, N.Y. |
| W. A. PHREANER | Philadelphia, Pa. |
| CHAS. E. PIKE | Philadelphia, Pa. |
| W. E. PINKHAM | New York, N.Y. |
| W. H. POTTER | Boston, Mass. |
| CHAS. P. PRUYN | Chicago, Ill. |
| H. C. REGISTER | Philadelphia, Pa. |
| F. A. ROY | New Orleans, La. |
| Z. T. SAILER | New York, N.Y. |
| L. D. SHEPARD | Boston, Mass. |
| GEO. R. SHIDLE | Pittsburg, Pa. |
| EUGENE H. SMITH | Boston, Mass. |
| B. HOLLY SMITH | Baltimore, Md. |
| H. A. SMITH | Cincinnati, Ohio. |
| S. G. STEVENS | Boston, Mass. |
| W. X. SUDDUTH | Minneapolis, Minn. |
| EUGENE S. TALBOTT | Chicago, Ill. |
| AMBLER TEES | Philadelphia, Pa. |
| J. D. THOMAS | Philadelphia, Pa. |
| JAMES TRUMAN | Philadelphia, Pa. |
| WM. H. TRUEMAN | Philadelphia, Pa. |
| W. W. WALKER | New York, N.Y. |
| I. F. WARDWELL | Stanford, Conn. |
| G. W. WARREN | Philadelphia, Pa. |
| T. S. WATERS | Baltimore, Md. |
| GEO. W. WELD | New York, N.Y. |
| JULIUS G. W. WERNER | Boston, Mass. |
| JACOB L. WILLIAMS | Boston, Mass. |
| R. B. WINDER | Baltimore, Md. |
| C. A. WOODWARD | New York, N.Y. |
| J. A. WOODWARD | Philadelphia, Pa. |
| W. A. WOODWARD | New York, N.Y. |
| W. J. YOUNGER | San Francisco, Cal. |

The officers of the INTERNATIONAL DENTAL PUBLICATION COMPANY serve without salary, the investments of the stockholders merely guarantee the success of the enterprise, ALL PROFITS being devoted to the enlargement or improvement of the INTERNATIONAL DENTAL JOURNAL.

**The support of every dentist having the welfare of his profession at heart is earnestly solicited. Subscription, $2.50 per Annum.**

*THE INTERNATIONAL DENTAL PUBLICATION CO.*

SUBSCRIBE FOR THE

### International Dental Journal,

EDITED BY JAMES TRUMAN, D.D.S.

### Published by the International Dental Publication Company.

LOUIS JACK, PRESIDENT, Philadelphia.
BENJAMIN LORD, VICE-PRESIDENT, New York.
C. N. PEIRCE, TREASURER, Philadelphia.
GEO. S. ALLAN, SECRETARY, 51 West Thirty-seventh St., New York.

**Publication Office, 716 Filbert St., Philadelphia, Pa.**

### STOCKHOLDERS.

| | |
|---|---|
| Geo. S. Allan | New York, N.Y. |
| R. R. Andrews | Cambridge, Mass. |
| H. A. Baker | Boston, Mass. |
| A. E. Baldwin | Chicago, Ill. |
| W. C. Barrett | Buffalo, N.Y. |
| S. T. Beale, Jr. | Philadelphia, Pa. |
| C. S. Beck | Wilkesbarre, Pa. |
| G. V. Black | Jacksonville, Ill. |
| C. F. W. Bodecker | New York, N.Y. |
| E. A. Bogue | New York, N.Y. |
| W. G. A. Bonwill | Philadelphia, Pa. |
| C. A. Brackett | Newport, R.I. |
| Thomas Bradley | New York, N.Y. |
| Edward C. Briggs | Boston, Mass. |
| Albert H. Brockway | Brooklyn, N.Y. |
| A. E. Brown | Chicago, Ill. |
| Wm. Carr | New York, N.Y. |
| Geo. H. Chance | Portland, Oregon. |
| G. F. Cheney | St. Johnsbury, Vt. |
| Dwight M. Clapp | Boston, Mass. |
| John T. Codman | Boston, Mass. |
| Chas. D. Cook | Brooklyn, N.Y. |
| J. N. Crouse | Chicago, Ill. |
| Edw. T. Darby | Philadelphia, Pa. |
| H. S. Draper | Boston, Mass. |
| Wm. H. Dwinelle | New York, N.Y. |
| A. R. Eaton | Elizabeth, N.J. |
| Albert T. Emery | Boston, Mass. |
| Chas. J. Essig | Philadelphia, Pa. |
| J. N. Farrer | New York, N.Y. |
| T. Fillebrown | Portland, Me. |
| W. B. Finney | Baltimore, Md. |
| T. A. Fletcher | New York, N.Y. |
| M. W. Foster | Baltimore, Md. |
| Chas. E. Francis | New York, N.Y. |
| E. C. Fundenberg | Pittsburg, Pa. |
| W. F. Fundenberg | Pittsburg, Pa. |
| W. H. Fundenberg | Pittsburg, Pa. |
| E. S. Gaylord | New Haven, Conn. |
| J. P. Geran | Brooklyn, N.Y. |
| A. H. Gilson | Boston, Mass. |
| S. H. Guilford | Philadelphia, Pa. |
| John I. Hart | New York, N.Y. |
| O. E. Hill | Brooklyn, N.Y. |
| J. F. P. Hodson | New York, N.Y. |
| J. Morgan Howe | New York, N.Y. |
| Robert Huey | Philadelphia, Pa. |
| A. O. Hunt | Iowa City, Iowa. |
| Louis Jack | Philadelphia, Pa |
| V. H. Jackson | New York, N.Y. |
| C. R. Jefferis | Wilmington, Del. |
| W. T. La Roche | Harrington Park, N.J. |
| W. K. Leech | Philadelphia Pa. |
| Wilbur F. Litch | Philadelphia, Pa. |
| J. Bond Littig | New York, N.Y. |
| Benjamin Lord | New York, N.Y. |
| Geo. W. Lovejoy | Montreal, Ont. |
| John S. Marshall | Chicago, Ill. |
| H. J. McKellops | St. Louis, Mo. |
| Charles McManus | Hartford, Conn. |
| James McManus | Hartford, Conn. |
| Dan'l Neal McQuillan | Philadelphia, Pa. |
| Frederic A. Merrill | Boston, Mass. |
| Horatio C. Meriam | Salem, Mass. |
| W. D. Miller | Berlin, Germany. |
| Geo. T. Moffatt | Boston, Mass. |
| A. L. Northrop | New York, N.Y. |
| W. E. Page | Boston, Mass. |
| S. B. Palmer | Syracuse, N.Y. |
| C. B. Parker | Brooklyn, N.Y. |
| D. D. Peabody | Stoneham, Mass. |
| C. N. Peirce | Philadelphia, Pa. |
| S. G. Perry | New York, N.Y. |
| W. A. Phreaner | Philadelphia, Pa. |
| Chas. E. Pike | Philadelphia, Pa. |
| W. E. Pinkham | New York, N.Y. |
| W. H. Potter | Boston, Mass. |
| Chas. P. Pruyn | Chicago, Ill. |
| H. C. Register | Philadelphia, Pa. |
| F. A. Roy | New Orleans, La. |
| Z. T. Sailer | New York, N.Y. |
| L. D. Shepard | Boston, Mass. |
| Geo. R. Shidle | Pittsburg, Pa. |
| Eugene H. Smith | Boston, Mass. |
| B. Holly Smith | Baltimore, Md. |
| H. A. Smith | Cincinnati, Ohio. |
| S. G. Stevens | Boston, Mass. |
| W. X. Sudduth | Minneapolis, Minn. |
| Eugene S. Talbott | Chicago, Ill. |
| Ambler Tees | Philadelphia, Pa. |
| J. D. Thomas | Philadelphia, Pa. |
| James Truman | Philadelphia, Pa. |
| Wm. H. Trueman | Philadelphia, Pa. |
| W. W. Walker | New York, N.Y. |
| I. F. Wardwell | Stanford, Conn. |
| G. W. Warren | Philadelphia, Pa. |
| T. S. Waters | Baltimore, Md. |
| Geo. W. Weld | New York, N.Y. |
| Julius G. W. Werner | Boston, Mass. |
| Jacob L. Williams | Boston, Mass. |
| R. B. Winder | Baltimore, Md. |
| C. A. Woodward | New York, N.Y. |
| J. A. Woodward | Philadelphia, Pa. |
| W. A. Woodward | New York, N.Y. |
| W. J. Younger | San Francisco, Cal. |

The officers of the INTERNATIONAL DENTAL PUBLICATION COMPANY serve without salary, the investments of the stockholders merely guarantee the success of the enterprise, ALL PROFITS being devoted to the enlargement or improvement of the INTERNATIONAL DENTAL JOURNAL.

**The support of every dentist having the welfare of his profession at heart is earnestly solicited. Subscription, $2.50 per Annum.**

*THE INTERNATIONAL DENTAL PUBLICATION CO.*

SUBSCRIBE FOR THE

### International Dental Journal,

EDITED BY JAMES TRUMAN, D.D.S.

### Published by the International Dental Publication Company.

LOUIS JACK, PRESIDENT, Philadelphia.
BENJAMIN LORD, VICE-PRESIDENT, New York.
C. N. PEIRCE, TREASURER, Philadelphia.
GEO. S. ALLAN, SECRETARY, 51 West Thirty-seventh St., New York.

**Publication Office, 716 Filbert St., Philadelphia, Pa.**

### STOCKHOLDERS.

| | |
|---|---|
| GEO. S. ALLAN | New York, N.Y. |
| R. R. ANDREWS | Cambridge, Mass. |
| H. A. BAKER | Boston, Mass. |
| A. E. BALDWIN | Chicago, Ill. |
| W. C. BARRETT | Buffalo, N.Y. |
| S. T. BEALE, JR. | Philadelphia, Pa. |
| C. S. BECK | Wilkesbarre, Pa. |
| G. V. BLACK | Jacksonville, Ill. |
| C. F. W. BODECKER | New York, N.Y. |
| E. A. BOGUE | New York, N.Y. |
| W. G. A. BONWILL | Philadelphia, Pa. |
| C. A. BRACKETT | Newport, R.I. |
| THOMAS BRADLEY | New York, N.Y. |
| EDWARD C. BRIGGS | Boston, Mass. |
| ALBERT H. BROCKWAY | Brooklyn, N.Y. |
| A. E. BROWN | Chicago, Ill. |
| WM. CARR | New York, N.Y. |
| GEO. H. CHANCE | Portland, Oregon. |
| G. F. CHENEY | St. Johnsbury, Vt. |
| DWIGHT M. CLAPP | Boston, Mass. |
| JOHN T. CODMAN | Boston, Mass. |
| CHAS. D. COOK | Brooklyn, N.Y. |
| J. N. CROUSE | Chicago, Ill. |
| EDW. T. DARBY | Philadelphia, Pa. |
| H. S. DRAPER | Boston, Mass. |
| WM. H. DWINELLE | New York, N.Y. |
| A. R. EATON | Elizabeth, N.J. |
| ALBERT T. EMERY | Boston, Mass. |
| CHAS. J. ESSIG | Philadelphia, Pa. |
| J. N. FARRER | New York, N.Y. |
| T. FILLEBROWN | Portland, Me. |
| W. B. FINNEY | Baltimore, Md. |
| T. A. FLETCHER | New York, N.Y. |
| M. W. FOSTER | Baltimore, Md. |
| CHAS. E. FRANCIS | New York, N.Y. |
| E. C. FUNDENBERG | Pittsburg, Pa. |
| W. F. FUNDENBERG | Pittsburg, Pa. |
| W. H. FUNDENBERG | Pittsburg, Pa. |
| E. S. GAYLORD | New Haven, Conn. |
| J. P. GERAN | Brooklyn, N.Y. |
| A. H. GILSON | Boston, Mass. |
| S. H. GUILFORD | Philadelphia, Pa. |
| JOHN I. HART | New York, N.Y. |
| O. E. HILL | Brooklyn, N.Y. |
| J. F. P. HODSON | New York, N.Y. |
| J. MORGAN HOWE | New York, N.Y. |
| ROBERT HUEY | Philadelphia, Pa. |
| A. O. HUNT | Iowa City, Iowa. |
| LOUIS JACK | Philadelphia, Pa. |
| V. H. JACKSON | New York, N.Y. |
| C. R. JEFFERIS | Wilmington, Del. |
| W. T. LA ROCHE | Harrington Park, N.J. |
| W. K. LEECH | Philadelphia, Pa. |
| WILBUR F. LITCH | Philadelphia, Pa. |
| J. BOND LITTIG | New York, N.Y. |
| BENJAMIN LORD | New York, N.Y. |
| GEO. W. LOVEJOY | Montreal, Ont. |
| JOHN S. MARSHALL | Chicago, Ill. |
| H. J. MCKELLOPS | St. Louis, Mo. |
| CHARLES MCMANUS | Hartford, Conn. |
| JAMES MCMANUS | Hartford, Conn. |
| DAN'L NEAL MCQUILLAN | Philadelphia, Pa. |
| FREDERIC A. MERRILL | Boston, Mass. |
| HORATIO C. MERIAM | Salem, Mass. |
| W. D. MILLER | Berlin, Germany. |
| GEO. T. MOFFATT | Boston, Mass. |
| A. L. NORTHROP | New York, N.Y. |
| W. E. PAGE | Boston, Mass. |
| S. B. PALMER | Syracuse, N.Y. |
| C. B. PARKER | Brooklyn, N.Y. |
| D. D. PEABODY | Stoneham, Mass. |
| C. N. PEIRCE | Philadelphia, Pa. |
| S. G. PERRY | New York, N.Y. |
| W. A. PHREANER | Philadelphia, Pa. |
| CHAS. E. PIKE | Philadelphia, Pa. |
| W. E. PINKHAM | New York, N.Y. |
| W. H. POTTER | Boston, Mass. |
| CHAS. P. PRUYN | Chicago, Ill. |
| H. C. REGISTER | Philadelphia, Pa. |
| F. A. ROY | New Orleans, La. |
| Z. T. SAILER | New York, N.Y. |
| L. D. SHEPARD | Boston, Mass. |
| GEO. R. SHIDLE | Pittsburg, Pa. |
| EUGENE H. SMITH | Boston, Mass. |
| B. HOLLY SMITH | Baltimore, Md. |
| H. A. SMITH | Cincinnati, Ohio. |
| S. G. STEVENS | Boston, Mass. |
| W. X. SUDDUTH | Minneapolis, Minn. |
| EUGENE S. TALBOTT | Chicago, Ill. |
| AMBLER TEES | Philadelphia, Pa. |
| J. D. THOMAS | Philadelphia, Pa. |
| JAMES TRUMAN | Philadelphia, Pa. |
| WM. H. TRUEMAN | Philadelphia, Pa. |
| W. W. WALKER | New York, N.Y. |
| I. F. WARDWELL | Stanford, Conn. |
| G. W. WARREN | Philadelphia, Pa. |
| T. S. WATERS | Baltimore, Md. |
| GEO. W. WELD | New York, N.Y. |
| JULIUS G. W. WERNER | Boston, Mass. |
| JACOB L. WILLIAMS | Boston, Mass. |
| R. B. WINDER | Baltimore, Md. |
| C. A. WOODWARD | New York, N.Y. |
| J. A. WOODWARD | Philadelphia, Pa. |
| W. A. WOODWARD | New York, N.Y. |
| W. J. YOUNGER | San Francisco, Cal. |

The officers of the INTERNATIONAL DENTAL PUBLICATION COMPANY serve without salary, the investments of the stockholders merely guarantee the success of the enterprise, ALL PROFITS being devoted to the enlargement or improvement of the INTERNATIONAL DENTAL JOURNAL.

**The support of every dentist having the welfare of his profession at heart is earnestly solicited. Subscription, $2.50 per Annum.**

*THE INTERNATIONAL DENTAL PUBLICATION CO.*

SUBSCRIBE FOR THE

### International Dental Journal,

EDITED BY JAMES TRUMAN, D.D.S.

**Published by the International Dental Publication Company.**

LOUIS JACK, PRESIDENT, Philadelphia.
BENJAMIN LORD, VICE-PRESIDENT, New York.
C. N. PEIRCE, TREASURER, Philadelphia.
GEO. S. ALLAN, SECRETARY, 51 West Thirty-seventh St., New York.

**Publication Office, 716 Filbert St., Philadelphia, Pa.**

STOCKHOLDERS.

| | |
|---|---|
| GEO. S. ALLAN | New York, N.Y. |
| R. R. ANDREWS | Cambridge, Mass. |
| H. A. BAKER | Boston, Mass. |
| A. E. BALDWIN | Chicago, Ill. |
| W. C. BARRETT | Buffalo, N.Y. |
| S. T. BEALE, JR. | Philadelphia, Pa. |
| C. S. BECK | Wilkesbarre, Pa. |
| G. V. BLACK | Jacksonville, Ill. |
| C. F. W. BODECKER | New York, N.Y. |
| E. A. BOGUE | New York, N.Y. |
| W. G. A. BONWILL | Philadelphia, Pa. |
| C. A. BRACKETT | Newport, R.I. |
| THOMAS BRADLEY | New York, N.Y. |
| EDWARD C. BRIGGS | Boston, Mass. |
| ALBERT H. BROCKWAY | Brooklyn, N.Y. |
| A. E. BROWN | Chicago, Ill. |
| WM. CARR | New York, N.Y. |
| GEO. H. CHANCE | Portland, Oregon. |
| G. F. CHENEY | St. Johnsbury, Vt. |
| DWIGHT M. CLAPP | Boston, Mass. |
| JOHN T. CODMAN | Boston, Mass. |
| CHAS. D. COOK | Brooklyn, N.Y. |
| J. N. CROUSE | Chicago, Ill. |
| EDW. T. DARBY | Philadelphia, Pa. |
| H. S. DRAPER | Boston, Mass. |
| WM. H. DWINELLE | New York, N.Y. |
| A. R. EATON | Elizabeth, N.J. |
| ALBERT T. EMERY | Boston, Mass. |
| CHAS. J. ESSIG | Philadelphia, Pa. |
| J. N. FARRER | New York, N.Y. |
| T. FILLEBROWN | Portland, Me. |
| W. B. FINNEY | Baltimore, Md. |
| T. A. FLETCHER | New York, N.Y. |
| M. W. FOSTER | Baltimore, Md. |
| CHAS. E. FRANCIS | New York, N.Y. |
| E. C. FUNDENBERG | Pittsburg, Pa. |
| W. F. FUNDENBERG | Pittsburg, Pa. |
| W. H. FUNDENBERG | Pittsburg, Pa. |
| E. S. GAYLORD | New Haven, Conn. |
| J. P. GERAN | Brooklyn, N.Y. |
| A. H. GILSON | Boston, Mass. |
| S. H. GUILFORD | Philadelphia, Pa. |
| JOHN I. HART | New York, N.Y. |
| O. E. HILL | Brooklyn, N.Y. |
| J. F. P. HODSON | New York, N.Y. |
| J. MORGAN HOWE | New York, N.Y. |
| ROBERT HUEY | Philadelphia, Pa. |
| A. O. HUNT | Iowa City, Iowa. |
| LOUIS JACK | Philadelphia, Pa |
| V. H. JACKSON | New York, N.Y. |
| C. R. JEFFERIS | Wilmington, Del. |
| W. T. LA ROCHE | Harrington Park, N.J. |
| W. K. LEECH | Philadelphia Pa. |
| WILBUR F. LITCH | Philadelphia, Pa. |
| J. BOND LITTIG | New York, N.Y. |
| BENJAMIN LORD | New York, N.Y. |
| GEO. W. LOVEJOY | Montreal, Ont. |
| JOHN S. MARSHALL | Chicago, Ill. |
| H. J. MCKELLOPS | St. Louis, Mo. |
| CHARLES MCMANUS | Hartford, Conn. |
| JAMES MCMANUS | Hartford, Conn. |
| DAN'L NEAL MCQUILLAN | Philadelphia, Pa. |
| FREDERIC A. MERRILL | Boston, Mass. |
| HORATIO C. MERIAM | Salem, Mass. |
| W. D. MILLER | Berlin, Germany. |
| GEO. T. MOFFATT | Boston, Mass. |
| A. L. NORTHROP | New York, N.Y. |
| W. E. PAGE | Boston, Mass. |
| S. B. PALMER | Syracuse, N.Y. |
| C. B. PARKER | Brooklyn, N.Y. |
| D. D. PEABODY | Stoneham, Mass. |
| C. N. PEIRCE | Philadelphia, Pa. |
| S. G. PERRY | New York, N.Y. |
| W. A. PHREANER | Philadelphia, Pa. |
| CHAS. E. PIKE | Philadelphia, Pa. |
| W. E. PINKHAM | New York, N.Y. |
| W. H. POTTER | Boston, Mass. |
| CHAS. P. PRUYN | Chicago, Ill. |
| H. C. REGISTER | Philadelphia, Pa. |
| F. A. ROY | New Orleans, La. |
| Z. T. SAILER | New York, N.Y. |
| L. D. SHEPARD | Boston, Mass. |
| GEO. R. SHIDLE | Pittsburg, Pa. |
| EUGENE H. SMITH | Boston, Mass. |
| B. HOLLY SMITH | Baltimore, Md. |
| H. A. SMITH | Cincinnati, Ohio. |
| S. G. STEVENS | Boston, Mass. |
| W. X. SUDDUTH | Minneapolis, Minn. |
| EUGENE S. TALBOTT | Chicago, Ill. |
| AMBLER TEES | Philadelphia, Pa. |
| J. D. THOMAS | Philadelphia, Pa. |
| JAMES TRUMAN | Philadelphia, Pa. |
| WM. H. TRUEMAN | Philadelphia, Pa. |
| W. W. WALKER | New York, N.Y. |
| I. F. WARDWELL | Stanford, Conn. |
| G. W. WARREN | Philadelphia, Pa. |
| T. S. WATERS | Baltimore, Md. |
| GEO. W. WELD | New York, N.Y. |
| JULIUS G. W. WERNER | Boston, Mass. |
| JACOB L. WILLIAMS | Boston, Mass. |
| R. B. WINDER | Baltimore, Md. |
| C. A. WOODWARD | New York, N.Y. |
| J. A. WOODWARD | Philadelphia, Pa. |
| W. A. WOODWARD | New York, N.Y. |
| W. J. YOUNGER | San Francisco, Cal. |

The officers of the INTERNATIONAL DENTAL PUBLICATION COMPANY serve without salary, the investments of the stockholders merely guarantee the success of the enterprise, ALL PROFITS being devoted to the enlargement or improvement of the INTERNATIONAL DENTAL JOURNAL.

**The support of every dentist having the welfare of his profession at heart is earnestly solicited. Subscription, $2.50 per Annum.**

*THE INTERNATIONAL DENTAL PUBLICATION CO.*

SUBSCRIBE FOR THE

### International Dental Journal,

EDITED BY JAMES TRUMAN, D.D.S.

### Published by the International Dental Publication Company.

LOUIS JACK, President, Philadelphia.
BENJAMIN LORD, Vice-President, New York.
C. N. PEIRCE, Treasurer, Philadelphia.
GEO. S. ALLAN, Secretary,
51 West Thirty-seventh St., New York.

**Publication Office, 716 Filbert St., Philadelphia, Pa.**

STOCKHOLDERS.

| | |
|---|---|
| Geo. S. Allan | New York, N.Y. |
| R. R. Andrews | Cambridge, Mass. |
| H. A. Baker | Boston, Mass. |
| A. E. Baldwin | Chicago, Ill. |
| W. C. Barrett | Buffalo, N.Y. |
| S. T. Beale, Jr. | Philadelphia, Pa. |
| C. S. Beck | Wilkesbarre, Pa. |
| G. V. Black | Jacksonville, Ill. |
| C. F. W. Bodecker | New York, N.Y. |
| E. A. Bogue | New York, N.Y. |
| W. G. A. Bonwill | Philadelphia, Pa. |
| C. A. Brackett | Newport, R.I. |
| Thomas Bradley | New York, N.Y. |
| Edward C. Briggs | Boston, Mass. |
| Albert H. Brockway | Brooklyn, N.Y. |
| A. E. Brown | Chicago, Ill. |
| Wm. Carr | New York, N.Y. |
| Geo. H. Chance | Portland, Oregon. |
| G. F. Cheney | St. Johnsbury, Vt. |
| Dwight M. Clapp | Boston, Mass. |
| John T. Codman | Boston, Mass. |
| Chas. D. Cook | Brooklyn, N.Y. |
| J. N. Crouse | Chicago, Ill. |
| Edw. T. Darby | Philadelphia, Pa. |
| H. S. Draper | Boston, Mass. |
| Wm. H. Dwinelle | New York, N.Y. |
| A. R. Eaton | Elizabeth, N.J. |
| Albert T. Emery | Boston, Mass. |
| Chas. J. Essig | Philadelphia, Pa. |
| J. N. Farrer | New York, N.Y. |
| T. Fillebrown | Portland, Me. |
| W. B. Finney | Baltimore, Md. |
| T. A. Fletcher | New York, N.Y. |
| M. W. Foster | Baltimore, Md. |
| Chas. E. Francis | New York, N.Y. |
| E. C. Fundenberg | Pittsburg, Pa. |
| W. F. Fundenberg | Pittsburg, Pa. |
| W. H. Fundenberg | Pittsburg, Pa. |
| E. S. Gaylord | New Haven, Conn. |
| J. P. Geran | Brooklyn, N.Y. |
| A. H. Gilson | Boston, Mass. |
| S. H. Guilford | Philadelphia, Pa. |
| John I. Hart | New York, N.Y. |
| O. E. Hill | Brooklyn, N.Y. |
| J. F. P. Hodson | New York, N.Y. |
| J. Morgan Howe | New York, N.Y. |
| Robert Huey | Philadelphia, Pa. |
| A. O. Hunt | Iowa City, Iowa. |
| Louis Jack | Philadelphia, Pa |
| V. H. Jackson | New York, N.Y. |
| C. R. Jefferis | Wilmington, Del. |
| W. T. La Roche | Harrington Park, N.J. |
| W. K. Leech | Philadelphia, Pa. |
| Wilbur F. Litch | Philadelphia, Pa. |
| J. Bond Littig | New York, N.Y. |
| Benjamin Lord | New York, N.Y. |
| Geo. W. Lovejoy | Montreal, Ont. |
| John S. Marshall | Chicago, Ill. |
| H. J. McKellops | St. Louis, Mo. |
| Charles McManus | Hartford, Conn. |
| James McManus | Hartford, Conn. |
| Dan'l Neal McQuillan | Philadelphia, Pa. |
| Frederic A. Merrill | Boston, Mass. |
| Horatio C. Meriam | Salem, Mass. |
| W. D. Miller | Berlin, Germany. |
| Geo. T. Moffatt | Boston, Mass. |
| A. L. Northrop | New York, N.Y. |
| W. E. Page | Boston, Mass. |
| S. B. Palmer | Syracuse, N.Y. |
| C. B. Parker | Brooklyn, N.Y. |
| D. D. Peabody | Stoneham, Mass. |
| C. N. Peirce | Philadelphia, Pa. |
| S. G. Perry | New York, N.Y. |
| W. A. Phreaner | Philadelphia, Pa. |
| Chas. E. Pike | Philadelphia, Pa. |
| W. E. Pinkham | New York, N.Y. |
| W. H. Potter | Boston, Mass. |
| Chas. P. Pruyn | Chicago, Ill. |
| H. C. Register | Philadelphia, Pa. |
| F. A. Roy | New Orleans, La. |
| Z. T. Sailer | New York, N.Y. |
| L. D. Shepard | Boston, Mass. |
| Geo. R. Shidle | Pittsburg, Pa. |
| Eugene H. Smith | Boston, Mass. |
| B. Holly Smith | Baltimore, Md. |
| H. A. Smith | Cincinnati, Ohio. |
| S. G. Stevens | Boston, Mass. |
| W. X. Sudduth | Minneapolis, Minn. |
| Eugene S. Talbott | Chicago, Ill. |
| Ambler Tees | Philadelphia, Pa. |
| J. D. Thomas | Philadelphia, Pa. |
| James Truman | Philadelphia, Pa. |
| Wm. H. Trueman | Philadelphia, Pa. |
| W. W. Walker | New York, N.Y. |
| I. F. Wardwell | Stanford, Conn. |
| G. W. Warren | Philadelphia, Pa. |
| T. S. Waters | Baltimore, Md. |
| Geo. W. Weld | New York, N.Y. |
| Julius G. W. Werner | Boston, Mass. |
| Jacob L. Williams | Boston, Mass. |
| R. B. Winder | Baltimore, Md. |
| C. A. Woodward | New York, N.Y. |
| J. A. Woodward | Philadelphia, Pa. |
| W. A. Woodward | New York, N.Y. |
| W. J. Younger | San Francisco, Cal. |

The officers of the INTERNATIONAL DENTAL PUBLICATION COMPANY serve without salary, the investments of the stockholders merely guarantee the success of the enterprise, ALL PROFITS being devoted to the enlargement or improvement of the INTERNATIONAL DENTAL JOURNAL.

**The support of every dentist having the welfare of his profession at heart is earnestly solicited. Subscription, $2.50 per Annum.**

*THE INTERNATIONAL DENTAL PUBLICATION CO.*

SUBSCRIBE FOR THE

### International Dental Journal,

EDITED BY JAMES TRUMAN, D.D.S.

### Published by the International Dental Publication Company.

LOUIS JACK, PRESIDENT, Philadelphia.
BENJAMIN LORD, VICE-PRESIDENT, New York.
C. N. PEIRCE, TREASURER, Philadelphia.
GEO. S. ALLAN, SECRETARY, 51 West Thirty-seventh St., New York.

**Publication Office, 716 Filbert St., Philadelphia, Pa.**

STOCKHOLDERS.

| | |
|---|---|
| Geo. S. Allan | New York, N.Y. |
| R. R. Andrews | Cambridge, Mass. |
| H. A. Baker | Boston, Mass. |
| A. E. Baldwin | Chicago, Ill. |
| W. C. Barrett | Buffalo, N.Y. |
| S. T. Beale, Jr. | Philadelphia, Pa. |
| C. S. Beck | Wilkesbarre, Pa. |
| G. V. Black | Jacksonville, Ill. |
| C. F. W. Bodecker | New York, N.Y. |
| E. A. Bogue | New York, N.Y. |
| W. G. A. Bonwill | Philadelphia, Pa. |
| C. A. Brackett | Newport, R.I. |
| Thomas Bradley | New York, N.Y. |
| Edward C. Briggs | Boston, Mass. |
| Albert H. Brockway | Brooklyn, N.Y. |
| A. E. Brown | Chicago, Ill. |
| Wm. Carr | New York, N.Y. |
| Geo. H. Chance | Portland, Oregon. |
| G. F. Cheney | St. Johnsbury, Vt. |
| Dwight M. Clapp | Boston, Mass. |
| John T. Codman | Boston, Mass. |
| Chas. D. Cook | Brooklyn, N.Y. |
| J. N. Crouse | Chicago, Ill. |
| Edw. T. Darby | Philadelphia, Pa. |
| H. S. Draper | Boston, Mass. |
| Wm. H. Dwinelle | New York, N.Y. |
| A. R. Eaton | Elizabeth, N.J. |
| Albert T. Emery | Boston, Mass. |
| Chas. J. Essig | Philadelphia, Pa. |
| J. N. Farrer | New York, N.Y. |
| T. Fillebrown | Portland, Me. |
| W. B. Finney | Baltimore, Md. |
| T. A. Fletcher | New York, N.Y. |
| M. W. Foster | Baltimore, Md. |
| Chas. E. Francis | New York, N.Y. |
| E. C. Fundenberg | Pittsburg, Pa. |
| W. F. Fundenberg | Pittsburg, Pa. |
| W. H. Fundenberg | Pittsburg, Pa. |
| E. S. Gaylord | New Haven, Conn. |
| J. P. Geran | Brooklyn, N.Y. |
| A. H. Gilson | Boston, Mass. |
| S. H. Guilford | Philadelphia, Pa. |
| John I. Hart | New York, N.Y. |
| O. E. Hill | Brooklyn, N.Y. |
| J. F. P. Hodson | New York, N.Y. |
| J. Morgan Howe | New York, N.Y. |
| Robert Huey | Philadelphia, Pa. |
| A. O. Hunt | Iowa City, Iowa. |
| Louis Jack | Philadelphia, Pa |
| V. H. Jackson | New York, N.Y. |
| C. R. Jefferis | Wilmington, Del. |
| W. T. La Roche | Harrington Park, N.J. |
| W. K. Leech | Philadelphia, Pa. |
| Wilbur F. Litch | Philadelphia, Pa. |
| J. Bond Littig | New York, N.Y. |
| Benjamin Lord | New York, N.Y. |
| Geo. W. Lovejoy | Montreal, Ont. |
| John S. Marshall | Chicago, Ill. |
| H. J. McKellops | St. Louis, Mo. |
| Charles McManus | Hartford, Conn. |
| James McManus | Hartford, Conn. |
| Dan'l Neal McQuillan | Philadelphia, Pa. |
| Frederic A. Merrill | Boston, Mass. |
| Horatio C. Meriam | Salem, Mass. |
| W. D. Miller | Berlin, Germany. |
| Geo. T. Moffatt | Boston, Mass. |
| A. L. Northrop | New York, N.Y. |
| W. E. Page | Boston, Mass. |
| S. B. Palmer | Syracuse, N.Y. |
| C. B. Parker | Brooklyn, N.Y. |
| D. D. Peabody | Stoneham, Mass. |
| C. N. Peirce | Philadelphia, Pa. |
| S. G. Perry | New York, N.Y. |
| W. A. Phreaner | Philadelphia, Pa. |
| Chas. E. Pike | Philadelphia, Pa. |
| W. E. Pinkham | New York, N.Y. |
| W. H. Potter | Boston, Mass. |
| Chas. P. Pruyn | Chicago, Ill. |
| H. C. Register | Philadelphia, Pa. |
| F. A. Roy | New Orleans, La. |
| Z. T. Sailer | New York, N.Y. |
| L. D. Shepard | Boston, Mass. |
| Geo. R. Shidle | Pittsburg, Pa. |
| Eugene H. Smith | Boston, Mass. |
| B. Holly Smith | Baltimore, Md. |
| H. A. Smith | Cincinnati, Ohio. |
| S. G. Stevens | Boston, Mass. |
| W. X. Sudduth | Minneapolis, Minn. |
| Eugene S. Talbott | Chicago, Ill. |
| Ambler Tees | Philadelphia, Pa. |
| J. D. Thomas | Philadelphia, Pa. |
| James Truman | Philadelphia, Pa. |
| Wm. H. Trueman | Philadelphia, Pa. |
| W. W. Walker | New York, N.Y. |
| I. F. Wardwell | Stanford, Conn. |
| G. W. Warren | Philadelphia, Pa. |
| T. S. Waters | Baltimore, Md. |
| Geo. W. Weld | New York, N.Y. |
| Julius G. W. Werner | Boston, Mass. |
| Jacob L. Williams | Boston, Mass. |
| R. B. Winder | Baltimore, Md. |
| C. A. Woodward | New York, N.Y. |
| J. A. Woodward | Philadelphia, Pa. |
| W. A. Woodward | New York, N.Y. |
| W. J. Younger | San Francisco, Cal. |

The officers of the INTERNATIONAL DENTAL PUBLICATION COMPANY serve without salary, the investments of the stockholders merely guarantee the success of the enterprise, ALL PROFITS being devoted to the enlargement or improvement of the INTERNATIONAL DENTAL JOURNAL.

**The support of every dentist having the welfare of his profession at heart is earnestly solicited. Subscription, $2.50 per Annum.**

*THE INTERNATIONAL DENTAL PUBLICATION CO.*

SUBSCRIBE FOR THE

### International Dental Journal,

EDITED BY JAMES TRUMAN, D.D.S.

**Published by the International Dental Publication Company.**

LOUIS JACK, PRESIDENT, Philadelphia.
BENJAMIN LORD, VICE-PRESIDENT, New York.
C. N. PEIRCE, TREASURER, Philadelphia.
GEO. S. ALLAN, SECRETARY, 51 West Thirty-seventh St., New York.

**Publication Office, 716 Filbert St., Philadelphia, Pa.**

### STOCKHOLDERS.

| | |
|---|---|
| GEO. S. ALLAN | New York, N.Y. |
| R. R. ANDREWS | Cambridge, Mass. |
| H. A. BAKER | Boston, Mass. |
| A. E. BALDWIN | Chicago, Ill. |
| W. C. BARRETT | Buffalo, N.Y. |
| S. T. BEALE, JR. | Philadelphia, Pa. |
| C. S. BECK | Wilkesbarre, Pa. |
| G. V. BLACK | Jacksonville, Ill. |
| C. F. W. BODECKER | New York, N.Y. |
| E. A. BOGUE | New York, N.Y. |
| W. G. A. BONWILL | Philadelphia, Pa. |
| C. A. BRACKETT | Newport, R.I. |
| THOMAS BRADLEY | New York, N.Y. |
| EDWARD C. BRIGGS | Boston, Mass. |
| ALBERT H. BROCKWAY | Brooklyn, N.Y. |
| A. E. BROWN | Chicago, Ill. |
| WM. CARR | New York, N.Y. |
| GEO. H. CHANCE | Portland, Oregon. |
| G. F. CHENEY | St. Johnsbury, Vt. |
| DWIGHT M. CLAPP | Boston, Mass. |
| JOHN T. CODMAN | Boston, Mass. |
| CHAS. D. COOK | Brooklyn, N.Y. |
| J. N. CROUSE | Chicago, Ill. |
| EDW. T. DARBY | Philadelphia, Pa. |
| H. S. DRAPER | Boston, Mass. |
| WM. H. DWINELLE | New York, N.Y. |
| A. R. EATON | Elizabeth, N.J. |
| ALBERT T. EMERY | Boston, Mass. |
| CHAS. J. ESSIG | Philadelphia, Pa. |
| J. N. FARRER | New York, N.Y. |
| T. FILLEBROWN | Portland, Me. |
| W. B. FINNEY | Baltimore, Md. |
| T. A. FLETCHER | New York, N.Y. |
| M. W. FOSTER | Baltimore, Md. |
| CHAS. E. FRANCIS | New York, N.Y. |
| E. C. FUNDENBERG | Pittsburg, Pa. |
| W. F. FUNDENBERG | Pittsburg, Pa. |
| W. H. FUNDENBERG | Pittsburg, Pa. |
| E. S. GAYLORD | New Haven, Conn. |
| J. P. GERAN | Brooklyn, N.Y. |
| A. H. GILSON | Boston, Mass. |
| S. H. GUILFORD | Philadelphia, Pa. |
| JOHN I. HART | New York, N.Y. |
| O. E. HILL | Brooklyn, N.Y. |
| J. F. P. HODSON | New York, N.Y. |
| J. MORGAN HOWE | New York, N.Y. |
| ROBERT HUEY | Philadelphia, Pa. |
| A. O. HUNT | Iowa City, Iowa. |
| LOUIS JACK | Philadelphia, Pa |
| V. H. JACKSON | New York, N.Y. |
| C. R. JEFFERIS | Wilmington, Del. |
| W. T. LA ROCHE | Harrington Park, N.J. |
| W. K. LEECH | Philadelphia, Pa. |
| WILBUR F. LITCH | Philadelphia, Pa. |
| J. BOND LITTIG | New York, N.Y. |
| BENJAMIN LORD | New York, N.Y. |
| GEO. W. LOVEJOY | Montreal, Ont. |
| JOHN S. MARSHALL | Chicago, Ill. |
| H. J. MCKELLOPS | St. Louis, Mo. |
| CHARLES MCMANUS | Hartford, Conn. |
| JAMES MCMANUS | Hartford, Conn. |
| DAN'L NEAL MCQUILLAN | Philadelphia, Pa. |
| FREDERIC A. MERRILL | Boston, Mass. |
| HORATIO C. MERIAM | Salem, Mass. |
| W. D. MILLER | Berlin, Germany. |
| GEO. T. MOFFATT | Boston, Mass. |
| A. L. NORTHROP | New York, N.Y. |
| W. E. PAGE | Boston, Mass. |
| S. B. PALMER | Syracuse, N.Y. |
| C. B. PARKER | Brooklyn, N.Y. |
| D. D. PEABODY | Stoneham, Mass. |
| C. N. PEIRCE | Philadelphia, Pa. |
| S. G. PERRY | New York, N.Y. |
| W. A. PHREANER | Philadelphia, Pa. |
| CHAS. E. PIKE | Philadelphia, Pa. |
| W. E. PINKHAM | New York, N.Y. |
| W. H. POTTER | Boston, Mass. |
| CHAS. P. PRUYN | Chicago, Ill. |
| H. C. REGISTER | Philadelphia, Pa. |
| F. A. ROY | New Orleans, La. |
| Z. T. SAILER | New York, N.Y. |
| L. D. SHEPARD | Boston, Mass. |
| GEO. R. SHIDLE | Pittsburg, Pa. |
| EUGENE H. SMITH | Boston, Mass. |
| B. HOLLY SMITH | Baltimore, Md. |
| H. A. SMITH | Cincinnati, Ohio. |
| S. G. STEVENS | Boston, Mass. |
| W. X. SUDDUTH | Minneapolis, Minn. |
| EUGENE S. TALBOTT | Chicago, Ill. |
| AMBLER TEES | Philadelphia, Pa. |
| J. D. THOMAS | Philadelphia, Pa. |
| JAMES TRUMAN | Philadelphia, Pa. |
| WM. H. TRUEMAN | Philadelphia, Pa. |
| W. W. WALKER | New York, N.Y. |
| I. F. WARDWELL | Stanford, Conn. |
| G. W. WARREN | Philadelphia, Pa. |
| T. S. WATERS | Baltimore, Md. |
| GEO. W. WELD | New York, N.Y. |
| JULIUS G. W. WERNER | Boston, Mass. |
| JACOB L. WILLIAMS | Boston, Mass. |
| R. B. WINDER | Baltimore, Md. |
| C. A. WOODWARD | New York, N.Y. |
| J. A. WOODWARD | Philadelphia, Pa. |
| W. A. WOODWARD | New York, N.Y. |
| W. J. YOUNGER | San Francisco, Cal. |

The officers of the INTERNATIONAL DENTAL PUBLICATION COMPANY serve without salary, the investments of the stockholders merely guarantee the success of the enterprise, ALL PROFITS being devoted to the enlargement or improvement of the INTERNATIONAL DENTAL JOURNAL.

**The support of every dentist having the welfare of his profession at heart is earnestly solicited. Subscription, $2.50 per Annum.**

***THE INTERNATIONAL DENTAL PUBLICATION CO.***

SUBSCRIBE FOR THE

### International Dental Journal,

EDITED BY JAMES TRUMAN, D.D.S.

### Published by the International Dental Publication Company.

LOUIS JACK, PRESIDENT, Philadelphia.
BENJAMIN LORD, VICE-PRESIDENT, New York.
C. N. PEIRCE, TREASURER, Philadelphia.
GEO. S. ALLAN, SECRETARY, 51 West Thirty-seventh St., New York.

**Publication Office, 716 Filbert St., Philadelphia, Pa.**

### STOCKHOLDERS.

| | |
|---|---|
| GEO. S. ALLAN | New York, N.Y. |
| R. R. ANDREWS | Cambridge, Mass. |
| H. A. BAKER | Boston, Mass. |
| A. E. BALDWIN | Chicago, Ill. |
| W. C. BARRETT | Buffalo, N.Y. |
| S. T. BEALE, JR. | Philadelphia, Pa. |
| C. S. BECK | Wilkesbarre, Pa. |
| G. V. BLACK | Jacksonville, Ill. |
| C. F. W. BODECKER | New York, N.Y. |
| E. A. BOGUE | New York, N.Y. |
| W. G. A. BONWILL | Philadelphia, Pa. |
| C. A. BRACKETT | Newport, R.I. |
| THOMAS BRADLEY | New York, N.Y. |
| EDWARD C. BRIGGS | Boston, Mass. |
| ALBERT H. BROCKWAY | Brooklyn, N.Y. |
| A. E. BROWN | Chicago, Ill. |
| WM. CARR | New York, N.Y. |
| GEO. H. CHANCE | Portland, Oregon. |
| G. F. CHENEY | St. Johnsbury, Vt. |
| DWIGHT M. CLAPP | Boston, Mass. |
| JOHN T. CODMAN | Boston, Mass. |
| CHAS. D. COOK | Brooklyn, N.Y. |
| J. N. CROUSE | Chicago, Ill. |
| EDW. T. DARBY | Philadelphia, Pa. |
| H. S. DRAPER | Boston, Mass. |
| WM. H. DWINELLE | New York, N.Y. |
| A. R. EATON | Elizabeth, N.J. |
| ALBERT T. EMERY | Boston, Mass. |
| CHAS. J. ESSIG | Philadelphia, Pa. |
| J. N. FARRER | New York, N.Y. |
| T. FILLEBROWN | Portland, Me. |
| W. B. FINNEY | Baltimore, Md. |
| T. A. FLETCHER | New York, N.Y. |
| M. W. FOSTER | Baltimore, Md. |
| CHAS. E. FRANCIS | New York, N.Y. |
| E. C. FUNDENBERG | Pittsburg, Pa. |
| W. F. FUNDENBERG | Pittsburg, Pa. |
| W. H. FUNDENBERG | Pittsburg, Pa. |
| E. S. GAYLORD | New Haven, Conn. |
| J. P. GERAN | Brooklyn, N.Y. |
| A. H. GILSON | Boston, Mass. |
| S. H. GUILFORD | Philadelphia, Pa. |
| JOHN I. HART | New York, N.Y. |
| O. E. HILL | Brooklyn, N.Y. |
| J. F. P. HODSON | New York, N.Y. |
| J. MORGAN HOWE | New York, N.Y. |
| ROBERT HUEY | Philadelphia, Pa. |
| A. O. HUNT | Iowa City, Iowa. |
| LOUIS JACK | Philadelphia, Pa. |
| V. H. JACKSON | New York, N.Y. |
| C. R. JEFFERIS | Wilmington, Del. |
| W. T. LA ROCHE | Harrington Park, N.J. |
| W. K. LEECH | Philadelphia, Pa. |
| WILBUR F. LITCH | Philadelphia, Pa. |
| J. BOND LITTIG | New York, N.Y. |
| BENJAMIN LORD | New York, N.Y. |
| GEO. W. LOVEJOY | Montreal, Ont. |
| JOHN S. MARSHALL | Chicago, Ill. |
| H. J. MCKELLOPS | St. Louis, Mo. |
| CHARLES MCMANUS | Hartford, Conn. |
| JAMES MCMANUS | Hartford, Conn. |
| DAN'L NEAL MCQUILLAN | Philadelphia, Pa. |
| FREDERIC A. MERRILL | Boston, Mass. |
| HORATIO C. MERIAM | Salem, Mass. |
| W. D. MILLER | Berlin, Germany. |
| GEO. T. MOFFATT | Boston, Mass. |
| A. L. NORTHROP | New York, N.Y. |
| W. E. PAGE | Boston, Mass. |
| S. B. PALMER | Syracuse, N.Y. |
| C. B. PARKER | Brooklyn, N.Y. |
| D. D. PEABODY | Stoneham, Mass. |
| C. N. PEIRCE | Philadelphia, Pa. |
| S. G. PERRY | New York, N.Y. |
| W. A. PHREANER | Philadelphia, Pa. |
| CHAS. E. PIKE | Philadelphia, Pa. |
| W. E. PINKHAM | New York, N.Y. |
| W. H. POTTER | Boston, Mass. |
| CHAS. P. PRUYN | Chicago, Ill. |
| H. C. REGISTER | Philadelphia, Pa. |
| F. A. ROY | New Orleans, La. |
| Z. T. SAILER | New York, N.Y. |
| L. D. SHEPARD | Boston, Mass. |
| GEO. R. SHIDLE | Pittsburg, Pa. |
| EUGENE H. SMITH | Boston, Mass. |
| B. HOLLY SMITH | Baltimore, Md. |
| H. A. SMITH | Cincinnati, Ohio. |
| S. G. STEVENS | Boston, Mass. |
| W. X. SUDDUTH | Minneapolis, Minn. |
| EUGENE S. TALBOTT | Chicago, Ill. |
| AMBLER TEES | Philadelphia, Pa. |
| J. D. THOMAS | Philadelphia, Pa. |
| JAMES TRUMAN | Philadelphia, Pa. |
| WM. H. TRUEMAN | Philadelphia, Pa. |
| W. W. WALKER | New York, N.Y. |
| I. F. WARDWELL | Stanford, Conn. |
| G. W. WARREN | Philadelphia, Pa. |
| T. S. WATERS | Baltimore, Md. |
| GEO. W. WELD | New York, N.Y. |
| JULIUS G. W. WERNER | Boston, Mass. |
| JACOB L. WILLIAMS | Boston, Mass. |
| R. B. WINDER | Baltimore, Md. |
| C. A. WOODWARD | New York, N.Y. |
| J. A. WOODWARD | Philadelphia, Pa. |
| W. A. WOODWARD | New York, N.Y. |
| W. J. YOUNGER | San Francisco, Cal. |

The officers of the INTERNATIONAL DENTAL PUBLICATION COMPANY serve without salary, the investments of the stockholders merely guarantee the success of the enterprise, ALL PROFITS being devoted to the enlargement or improvement of the INTERNATIONAL DENTAL JOURNAL.

**The support of every dentist having the welfare of his profession at heart is earnestly solicited. Subscription, $2.50 per Annum.**

*THE INTERNATIONAL DENTAL PUBLICATION CO.*

SUBSCRIBE FOR THE

### International Dental Journal,

EDITED BY **JAMES TRUMAN, D.D.S.**

### Published by the International Dental Publication Company.

LOUIS JACK, President, Philadelphia. C. N. PEIRCE, Treasurer, Philadelphia.
BENJAMIN LORD, Vice-President, New York. GEO. S. ALLAN, Secretary, 51 West Thirty-seventh St., New York.

**Publication Office, 716 Filbert St., Philadelphia, Pa.**

STOCKHOLDERS.

| | |
|---|---|
| Geo. S. Allan | New York, N.Y. |
| R. R. Andrews | Cambridge, Mass. |
| H. A. Baker | Boston, Mass. |
| A. E. Baldwin | Chicago, Ill. |
| W. C. Barrett | Buffalo, N.Y. |
| S. T. Beale, Jr. | Philadelphia, Pa. |
| C. S. Beck | Wilkesbarre, Pa. |
| G. V. Black | Jacksonville, Ill. |
| C. F. W. Bodecker | New York, N.Y. |
| E. A. Bogue | New York, N.Y. |
| W. G. A. Bonwill | Philadelphia, Pa. |
| C. A. Brackett | Newport, R.I. |
| Thomas Bradley | New York, N.Y. |
| Edward C. Briggs | Boston, Mass. |
| Albert H. Brockway | Brooklyn, N.Y. |
| A. E. Brown | Chicago, Ill. |
| Wm. Carr | New York, N.Y. |
| Geo. H. Chance | Portland, Oregon. |
| G. F. Cheney | St. Johnsbury, Vt. |
| Dwight M. Clapp | Boston, Mass. |
| John T. Codman | Boston, Mass. |
| Chas. D. Cook | Brooklyn, N.Y. |
| J. N. Crouse | Chicago, Ill. |
| Edw. T. Darby | Philadelphia, Pa. |
| H. S. Draper | Boston, Mass. |
| Wm. H. Dwinelle | New York, N.Y. |
| A. R. Eaton | Elizabeth, N.J. |
| Albert T. Emery | Boston, Mass. |
| Chas. J. Essig | Philadelphia, Pa. |
| J. N. Farrer | New York, N.Y. |
| T. Fillebrown | Portland, Me. |
| W. B. Finney | Baltimore, Md. |
| T. A. Fletcher | New York, N.Y. |
| M. W. Foster | Baltimore, Md. |
| Chas. E. Francis | New York, N.Y. |
| E. C. Fundenberg | Pittsburg, Pa. |
| W. F. Fundenberg | Pittsburg, Pa. |
| W. H. Fundenberg | Pittsburg, Pa. |
| E. S. Gaylord | New Haven, Conn. |
| J. P. Geran | Brooklyn, N.Y. |
| A. H. Gilson | Boston, Mass. |
| S. H. Guilford | Philadelphia, Pa. |
| John I. Hart | New York, N.Y. |
| O. E. Hill | Brooklyn, N.Y. |
| J. F. P. Hodson | New York, N.Y. |
| J. Morgan Howe | New York, N.Y. |
| Robert Huey | Philadelphia, Pa. |
| A. O. Hunt | Iowa City, Iowa. |
| Louis Jack | Philadelphia, Pa. |
| V. H. Jackson | New York, N.Y. |
| C. R. Jefferis | Wilmington, Del. |
| W. T. La Roche | Harrington Park, N.J. |
| W. K. Leech | Philadelphia, Pa. |
| Wilbur F. Litch | Philadelphia, Pa. |
| J. Bond Littig | New York, N.Y. |
| Benjamin Lord | New York, N.Y. |
| Geo. W. Lovejoy | Montreal, Ont. |
| John S. Marshall | Chicago, Ill. |
| H. J. McKellops | St. Louis, Mo. |
| Charles McManus | Hartford, Conn. |
| James McManus | Hartford, Conn. |
| Dan'l Neal McQuillan | Philadelphia, Pa. |
| Frederic A. Merrill | Boston, Mass. |
| Horatio C. Meriam | Salem, Mass. |
| W. D. Miller | Berlin, Germany. |
| Geo. T. Moffatt | Boston, Mass. |
| A. L. Northrop | New York, N.Y. |
| W. E. Page | Boston, Mass. |
| S. B. Palmer | Syracuse, N.Y. |
| C. B. Parker | Brooklyn, N.Y. |
| D. D. Peabody | Stoneham, Mass. |
| C. N. Peirce | Philadelphia, Pa. |
| S. G. Perry | New York, N.Y. |
| W. A. Phreaner | Philadelphia, Pa. |
| Chas. E. Pike | Philadelphia, Pa. |
| W. E. Pinkham | New York, N.Y. |
| W. H. Potter | Boston, Mass. |
| Chas. P. Pruyn | Chicago, Ill. |
| H. C. Register | Philadelphia, Pa. |
| F. A. Roy | New Orleans, La. |
| Z. T. Sailer | New York, N.Y. |
| L. D. Shepard | Boston, Mass. |
| Geo. R. Shidle | Pittsburg, Pa. |
| Eugene H. Smith | Boston, Mass. |
| B. Holly Smith | Baltimore, Md. |
| H. A. Smith | Cincinnati, Ohio. |
| S. G. Stevens | Boston, Mass. |
| W. X. Sudduth | Minneapolis, Minn. |
| Eugene S. Talbott | Chicago, Ill. |
| Ambler Tees | Philadelphia, Pa. |
| J. D. Thomas | Philadelphia, Pa. |
| James Truman | Philadelphia, Pa. |
| Wm. H. Trueman | Philadelphia, Pa. |
| W. W. Walker | New York, N.Y. |
| I. F. Wardwell | Stanford, Conn. |
| G. W. Warren | Philadelphia, Pa. |
| T. S. Waters | Baltimore, Md. |
| Geo. W. Weld | New York, N.Y. |
| Julius G. W. Werner | Boston, Mass. |
| Jacob L. Williams | Boston, Mass. |
| R. B. Winder | Baltimore, Md. |
| C. A. Woodward | New York, N.Y. |
| J. A. Woodward | Philadelphia, Pa. |
| W. A. Woodward | New York, N.Y. |
| W. J. Younger | San Francisco, Cal. |

The officers of the International Dental Publication Company serve without salary, the investments of the stockholders merely guarantee the success of the enterprise, ALL PROFITS being devoted to the enlargement or improvement of the International Dental Journal.

**The support of every dentist having the welfare of his profession at heart is earnestly solicited. Subscription, $2.50 per Annum.**

*THE INTERNATIONAL DENTAL PUBLICATION CO.*

